# Design, Synthesis and Bioactivity of Novel Low Bee-Toxicity Compounds Based on Flupyrimin

**DOI:** 10.3390/molecules27186133

**Published:** 2022-09-19

**Authors:** Xingxing Lu, Huan Xu, Xiaoming Zhang, Tengda Sun, Yufan Lin, Yongheng Zhang, Honghong Li, Xuesheng Li, Xinling Yang, Hongxia Duan, Yun Ling

**Affiliations:** 1 Innovation Center of Pesticide Research, Department of Applied Chemistry, College of Science, China Agricultural University, Beijing 100193, China; 2 Guangxi Key Laboratory of Agro-Environment and Agro-Product Safety, Agricultural College, Guangxi University, Nanning 530004, China

**Keywords:** low bee-toxicity, nicotinic insecticide, nicotinic acetylcholine receptor (nAChR), Flupyrimin derivatives, molecular docking

## Abstract

Neonicotinoids are important insecticides for controlling aphids in agriculture. Growing research suggested that neonicotinoid insecticides are a key factor causing the decline of global pollinator insects, such as bees. Flupyrimin (FLP) is a novel nicotinic insecticide with unique biological properties and no cross-resistance, and is safe for pollinators. Using FLP as the lead compound, a series of novel compounds were designed and synthesized by replacing the amide fragment with a sulfonamideone. Their structures were confirmed by ^1^H NMR, ^13^C NMR and HRMS spectra. Bioassay results showed that compound **2j** had good insecticidal activity against *Aphis glycines* with an LC_50_ value of 20.93 mg/L. Meanwhile, compound **2j** showed significantly lower acute oral and contact toxicity to *Apis mellifera*. In addition, compound **2j** interacted well with the protein in insect acetylcholine binding protein (AChBP). The molecular docking on honeybee nicotinic acetylcholine receptor (nAChR) indicated that the sulfonamide group of compound **2j** did not form a hydrogen bond with Arg173 of the β subunit, which conforms to the reported low bee-toxicity conformation. In general, target compound **2j** can be regarded as a bee-friendly insecticide candidate.

## 1. Introduction

**Figure 1:**
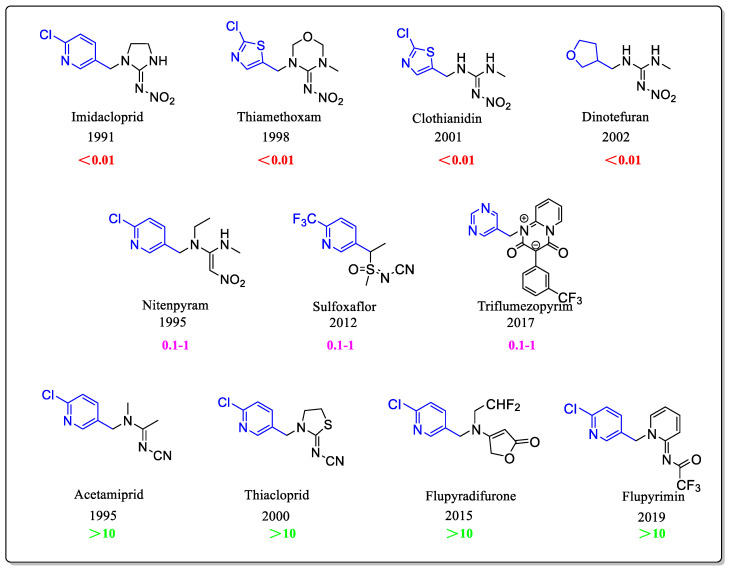
Insecticides targeting insect nicotinic acetylcholine receptors and their oral toxicity to bees (LD_50_, μg/bee).

Aphids are distributed worldwide and considered to be major agricultural pests, with about 250 species being the most destructive pests of cultivated plants [1]. Aphids are difficult to control because of their diverse species and the ability to reproduce rapidly. They can also survive in any part of the plant, consume a large amount of nutrients needed by plants and transmit more than 200 viral diseases. Furthermore, they have already emerged resistant to many pesticides [2,3,4]. Currently, the control of aphids still depends on chemical insecticides. Since the emergence of the commercial neonicotinoid imidacloprid (**IMI**) in 1991, neonicotinoid insecticides (Figure 1) rapidly became important insecticides to effectively control Hemiptera pests, such as aphids, leafhoppers, whiteflies, and so on, due to their unique lethal activity and low toxicity [5,6,7,8].

**Figure 2:**
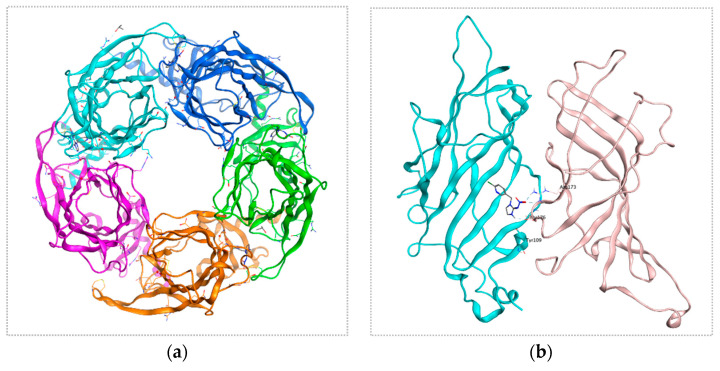
The model structure of nicotinic acetylcholine receptors ((**a**) insect AChBP; (**b**) honeybee nAChR).

Neonicotinoid insecticides act on insect nicotinic acetylcholine receptors (nAChRs). For a long time, the three-dimensional crystal structure of insect nAChRs was not successfully extracted and purified owing to its complex transmembrane structural characteristics. Fortunately, acetylcholine binding protein (AChBP) was widely used as a perfect replacement for the extracellular ligand-binding domain of nAChRs [9]. In recent years, an increasing number of crystal structures of AChBP complexes with different neonicotinoid insecticides was reported, such as **IMI** (Figure 2a; PDB ID: 3C79), thiacloprid, clothianidin, the substrate acetylcholine and natural nicotine, etc. [10,11,12]. These provided a powerful biological template for studying the interaction between neonicotinoid insecticides and nAChRs further. Based on these complexed crystal structures, a large number of insecticides were discovered and developed [13,14].

Unfortunately, the widespread use of neonicotinoid insecticides led to the decline of global pollinator insects, such as bees, the loss of biodiversity and ecosystem damage [15]. As shown in Figure 1, most neonicotinoid insecticides exhibit moderate and high bee toxicity and were, thus, limited or banned as a means of crop protection by some states. Therefore, further investigation into alternatives is becoming increasingly urgent [16]. In order to explore the bee-toxicity molecular mechanism of neonicotinoid insecticides, Duan et al. constructed a three-dimensional structural model of the functional subunit AmeIα8/ratβ2 of honeybee nAChR (Figure 2b); by analyzing the interaction between neonicotinoid insecticides and honeybee nAChR [17,18], they found that the unique nitro group was the most critical element conferring high bee toxicity due to its hydrogen bonds with residue Arg173 of the β chain in Amelα8/ratβ2.

In recent years, several alternative products with low bee toxicity were successfully developed, such as flupyradifurone (**FPF**) and Flupyrimin (**FLP**) [19,20,21,22,23] (Figure 1). Among them, **FLP**, a new nAChR antagonist developed by Meiji Seika Co., Ltd., has unique biological properties, no cross-resistance with major neonicotinoid insecticides and is especially safe for pollinators, such as bees [20].

**Scheme 1:**
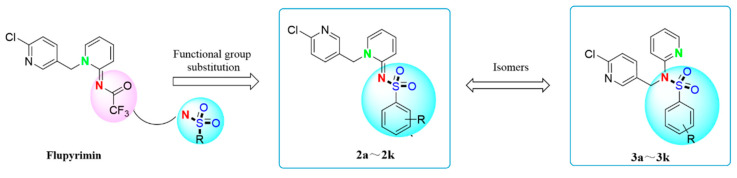
Design strategy of the target compounds.

Sulfonamide, present in the chemical structure of many medicine and pesticides, has excellent biological properties, including being anticancer, anti-inflammatory, insecticidal, antifungal and herbicidal [24,25,26,27]. In this paper, selecting **FLP** as the lead compound, the active fragment sulfonamide was introduced to replace the amide fragment (Scheme 1) to design and synthesize a total of 22 new compounds with two different isomers. The insecticidal activities against *Aphis glycines* and acute oral and contact toxicity against *Apis mellifera* of the target compounds were evaluated. Additionally, the binding mode of the compound with insect AchBP and honeybee nAChR were investigated by molecular docking.

## 2. Results and Discussion

### 2.1. Synthesis of Target Compounds

**Scheme 2:**
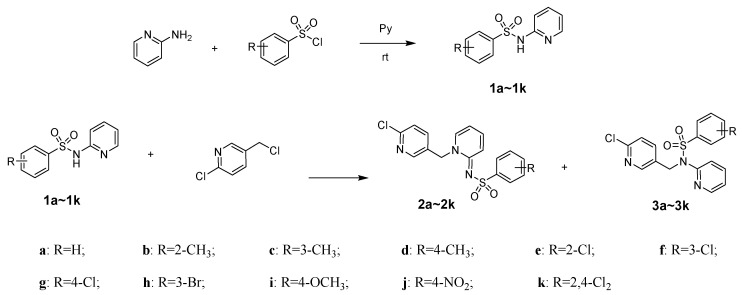
Synthetic routes of target compounds.

The synthetic routes of target compounds are shown in Scheme 2. The intermediate **1** was synthesized with 2-aminopyridine and benzenesulfonyl chloride. Two isomers were obtained from the reaction of intermediate **1** with 2-chloro-5-(chloromethyl)pyridine. As shown in Scheme 2, the difference between the formation of two sulfonamide moiety isomers, **2** and **3**, rests in the attachment of the sulfone moiety to the nitrogen atom. The formation of isomers **2** and **3** may be due to the nucleophilic attack by the ring nitrogen atom in the pyridine moiety and the amino group on the sulfonyl chloride. For this transformation, the key factors of the reaction and optimized reaction conditions were investigated. As shown in Table 1, different solvents (**entries 1–5**) influence the yield of sulfonamide moiety isomer **2a**, which can be obtained in the highest yield in DMF solution (**entry 5**). Through the optimization of conditions, it was found that different solvents and bases had no significant effect on the yield of isomer **3a**. In particular, **3a** cannot be obtained when using NaOH as the base. In summary, under room temperature conditions, in the presence of K_2_CO_3_ as the base in DMF solution, the desired sulfonamide product **2a** and **3a** were isolated at yields of 68.95% and 15.36%, respectively.

### 2.2. Structural Analysis

**Figure 3:**
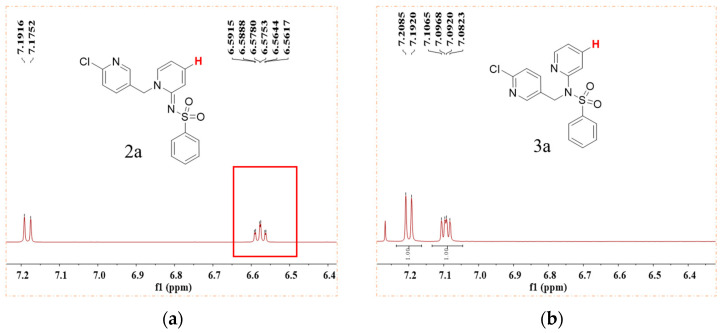
^1^H NMR spectra of compounds **2a** and **3a** ((**a**) **2a**; (**b**) **3a**).

**Figure 4:**
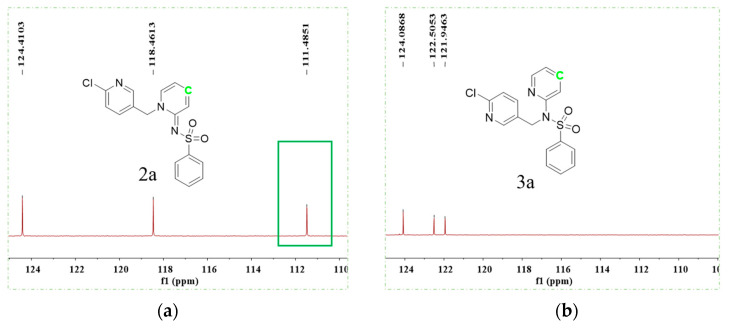
^13^C NMR spectra of compounds **2a** and **3a** ((**a**) **2a**; (**b**) **3a**).

The structures of the target compounds were confirmed by ^1^H NMR, ^13^C NMR and HRMS spectra (Figure S1). By analyzing the HRMS data, it was found that **2a**~**2k** and **3a**~**3k** were isomers. Further analysis found that the ^1^H NMR and ^13^C NMR data for the two isomers were different. Using **2a** and **3a** to illustrate: the aminopyridine ring of compound **2a** was rearranged due to the chloropyridine attached to the nitrogen atom of the aminopyridine ring; the chemical shifts of the hydrogen atoms and carbon atoms on the ring were, thus, moved to the high field. In ^1^H NMR, the proton-signal peak of the red hydrogen atom of compound **2a** was shown at *δ* 6.62 (Figure 3a), while the chemical shifts of all hydrogen atoms of **3a** were over *δ* 7 (Figure 3b). In ^13^C NMR, the proton-signal peak of the green carbon atom of compound **2a** was observed at *δ* 111.8 (Figure 4a), while the chemical shifts of all the carbon atoms of **3e** were over *δ* 118 (Figure 4b). These rules existed in all isomers.

**Figure 5:**
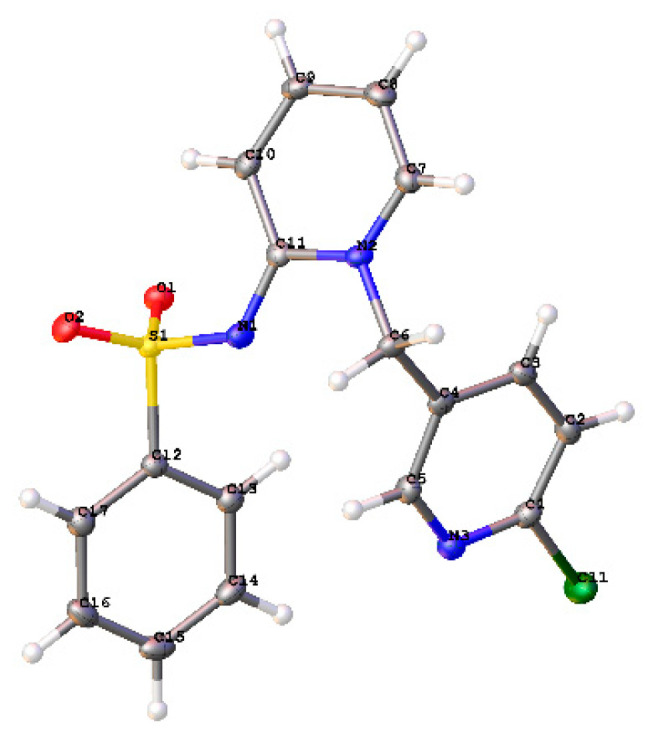
Crystal structure of compound **2a**.

In order to further confirm the configuration of the two isomers of the target compound, an X-ray single-crystal diffraction analysis was performed on target compound **2a** (Figure 5). CCDC No. 2,071,276 contains the supplementary crystallographic data for this paper. These data can be obtained free of charge via http://www.ccdc.cam.ac.uk/conts/retrieving.html accessed on 18 March 2020 (or from the CCDC, 12 Union Road, Cambridge CB2 1EZ, UK; Fax: +44-1223-336033; E-mail: deposit@ccdc.cam.ac.uk). The crystal structure of compound **2a** is shown in Figure 5. The X-ray diffraction analysis results showed that the 2-chloro-5-(chloromethyl) pyridine of compound **2a** was attached precisely to the nitrogen atom of the aminopyridine ring.

### 2.3. Insecticidal Activity against Adult Aphis glycines by the Target Compounds

Insecticidal activity against *A. glycines* by the target compounds was evaluated by the previously reported leaf-dipping method [28]. As shown in Table 2, some compounds had certain insecticidal activities at a concentration of 200 mg/L for 48 h. The insecticidal activities of compounds **2j**, **2g** and **3c** all exceeded 70.00%; in particular, compound **2j** reached 94.12%, which was better than that of pymetrozine (**PYM**: 81.62%). The structure–activity relationships of compounds **2a**~**2k** were also analyzed: several compounds with the electron-withdrawing group and halogen atoms, such as compounds **2j** (4-NO_2_), **2f** (3-Cl), **2g** (4-Cl) and **2h** (3-Br), showed better insecticidal activity than those with an electron-donating group, such as compounds **2b** (2-CH_3_), **2c**(3-CH_3_), **2d** (4-CH_3_) and **2i** (4-OCH_3_). Further analysis was performed on the different isomers of compounds **2** and **3**: when R was an electron-withdrawing group (Cl, Br, NO_2_), the insecticidal activity of isomers **2d**~**2k** was better than that of isomers **3d**~**3k**; when R was CH_3_, the insecticidal activity of isomers **3a**~**3c** was better than that of isomers **2a**~**2c**.

Subsequently, the LC_50_ values of several compounds with superior insecticidal activity against *A. glycines* were measured. As shown in Table 3, compounds **2g** and **2j** both showed better insecticidal activity than compound **3c**, especially the LC_50_ value of compound **2j** which was 20.93 mg/L, and which was slightly lower than that of **PYM** (9.98 mg/L). It was worth further research and optimization.

### 2.4. Oral and Contact Toxicity to Apis mellifera

In order to understand the potential selectivity of target compounds with the best insecticidal activity, the oral and contact toxicities to *A. mellifera* of compound **2j**, **FLP** and **IMI** were evaluated (Table 4) [29]. We were pleased to find that the lead compound **FLP** and target compound **2j** both show significantly lower acute oral and contact toxicity compared with **IMI**. Thus, compound **2j** was safe for *A. mellifera*. In general, the target compounds can be regarded as bee-friendly insecticidal candidates to be noted in the future.

### 2.5. Molecular Docking of Compounds with Nicotinic Acetylcholine Receptors

#### 2.5.1. Molecular Docking of Compounds with Insect AchBP

**Figure 6:**
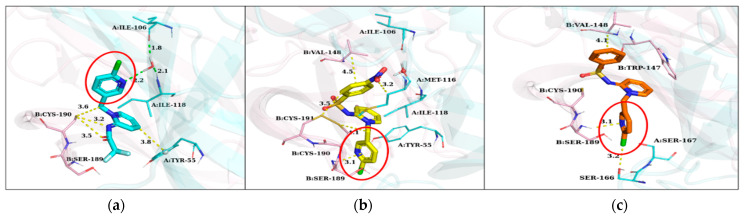
Docking of compounds **FLP** (**a**), **2j** (**b**) and **2a** (**c**) with insect AChBP.

In order to study the binding mode of compound **2j** with insect AchBP, molecular docking was performed. It was seen that compounds **2j** and **2a** both show a similar binding site to, but a different binding mode from, that of **FLP**. As shown in Figure 6a, **FLP** was found to generate strong hydrogen bonds with residues Ile106 and Ile118 through the water bridge formed by H_2_O419. Further analysis (Figure 6b,c) showed that the conformation of **2j** and **2a** were flipped, which caused the lack of a key water bridge interaction between the chloropyridine site (red circle) and the surrounding H_2_O419 for **FLP**. Fortunately, the oxygen atom of the nitro group in compound **2j**, with its high insecticidal activity, was found to form a hydrogen-bond force with the other residue, Met116, in insect AchBP; however, compound **2a**, with its low insecticidal activity, did not form any hydrogen bonds with several key residues, i.e., Ile106, Ile118 or Met116. This may also be the reason why compound **2j** maintains a certain insecticidal activity. Therefore, these residues, Ile106, Ile118 and Met116, may be the key amino acids binding to highly bioactive present target compounds. Preserving these key interactions between the target compounds with insect AchBP can provide a reference point for further enhancing the insecticidal activity of the compounds.

#### 2.5.2. Molecular Docking of Compounds with Honeybee nAChR Subunits AmeIα8/ratβ2

**Figure 7:**
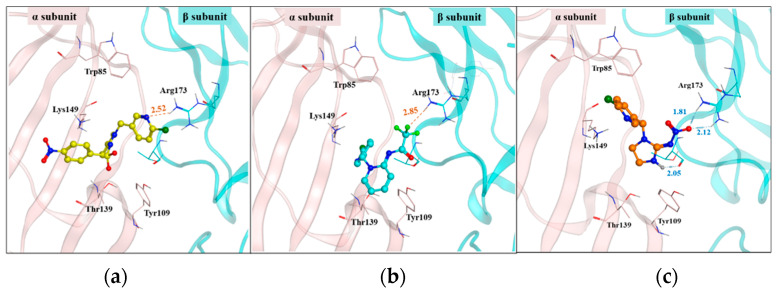
Docking of **2j** (**a**), **FLP** (**b**) and **IMI** (**c**) with honeybee subunits AmeIα_8_/ratβ_2_.

To further illustrate the molecular mechanism underlying the low bee-toxicity of target compounds, compound **2j** (which had high insecticidal activity against *A. glycines*) was also docked into the previously reported 3D model of the functional subunit to honeybee nAChR (AmeIα8/ratβ2) [18]. As shown in Figure 7, the binding mode of compound **2j** and **FLP** to honeybee nAChR is clearly different from that of high bee-toxicity **IMI**. In **IMI**, the key active fragment nitro site forms a hydrogen bond with the Arg173 residue of the β subunit of AmeIα8/ratβ2, which was reported to be a high honeybee-toxicity binding mode in a previous study [18]. However, the key active parts of compound **2j** and **FLP** were close to the α subunit and not bound to any residue of the β subunit. The closest distance of compound **2j** and **FLP** with bee nAChR subunits Arg173 were 2.52 Å and 2.85 Å, which are too weak to form hydrogen bonds. This may be why compound **2j** and **FLP** have low bee-toxicity. Therefore, the target compounds are proposed as potentially eco-friendly alternatives to insecticide and require further attention in the future.

### 2.6. DFT Calculation of Compounds ***2j*** and **FLP**

**Figure 8:**
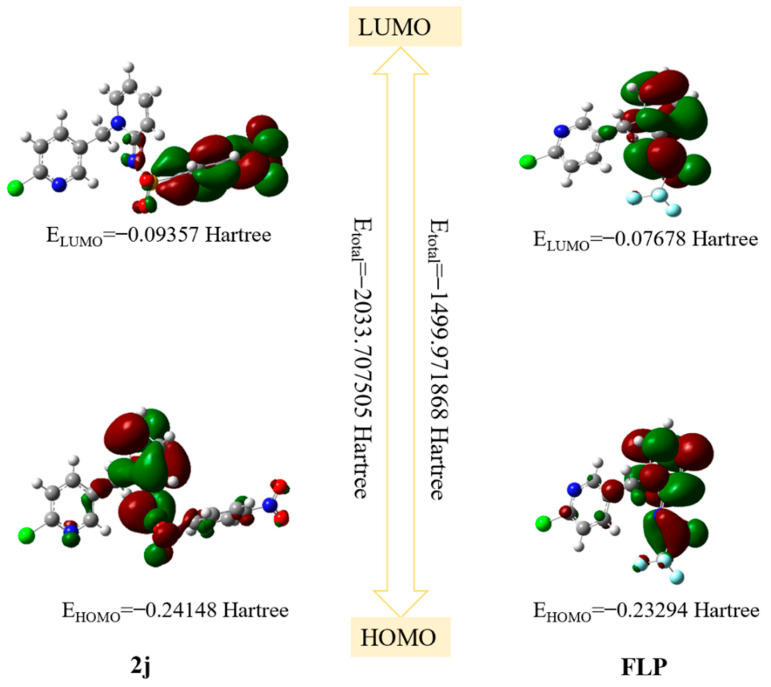
HOMO and LUMO of compounds **2j** and **FLP**.

DFT calculations of molecules were reported to be helpful for the design of more potent pesticides [31]. To find the structural features of the sulfonamide compound, the DFT calculation was carried out using the B3LYP method. Figure 8 shows that the geometries of compounds **2j** and **FLP** contain three parts: a chlorpyridine ring, 2-aminopyridine ring and sulfonamide group or amide group. The **2j** and **FLP** HOMO were mainly located on the 2-aminopyridine ring. While the **2j** LUMO was located on the benzenesulfonamide group, the **FLP** was still located on the 2-aminopyridine ring. It was found that the electron transition of compound **2j** occurred from the 2-aminopyridine ring to the benzenesulfonamide group via the newly included sulfonamide bridge. The total energies of the two compounds were different: compound **2j** (−2033.708 Hartree) had a lower value than compound FLP (−1499.972 Hartree). Analyzing the results of the DFT calculation, we guessed the target compounds with a higher total energy but without electron transition might exhibit better activity. The combination of the DFT results provided meaningful clues on the structural features of this new family of nicotinic insecticides, which may help in the design of more potent compounds.

## 3. Materials and Methods

### 3.1. Chemicals and Target Compounds

Bruker AVANCE NEO 500MHz nuclear magnetic resonance spectrometer (with TMS as internal standard, DMSO-*d*6 and CDCl_3_ as solvents); Agilent LC1200/MS Q-TOF6520 liquid mass spectrometer; Gemini E single crystal diffractometer and an X-4 precision microscope melting-point tester were used. Soybean aphids were raised in the laboratory. The column chromatography silica gel (200–300 mesh) and TLC plates used in this experiment were produced by Qingdao Ocean Chemical Co., Ltd. The reagents used were all commercially available analytical or chemically pure reagents. Unless otherwise specified, the solvents used were not treated with water.

#### 3.1.1. Preparation of N-(pyridin-2-yl) Substituted Benzene Sulfonamide

Taking the synthesis of **1a** as an example: a solution of benzenesulfonyl chloride (2.12 g, 12 mmol) was slowly added under an ice bath to a solution of 2-aminopyridine (0.94 g, 10 mmol) in pyridine. The mixture was stirred at room temperature for 1 h. Ice water was added and the mixture was stirred vigorously until a large amount of solid was precipitated. After filtration, the crude product **1a** was obtained with yellow solid (yield 73.0%) [32]. ^1^H NMR (500 MHz, Chloroform-*d*) *δ* 14.11 (s, 1H), 8.30 (d, *J* = 6.0 Hz, 1H), 7.86 (d, *J* = 7.7 Hz, 2H), 7.60 (s, 1H), 7.48–7.31 (m, 4H), 6.73 (s, 1H).

Intermediates **1a**~**1k** were prepared in the same way; all intermediates were known compounds [33].

#### 3.1.2. Preparation of (E)-N-(1-((6-chloropyridin-3-yl)methyl)pyridin-2(1H)-ylidene)benzenesulfonamide (**2a**) and N-((6-chloropyridin-3-yl)methyl)-N-(pyridin-2-yl)benzenesulfonamide (**3a**) 

Taking the synthesis of **2a** and **3a** as an example: the intermediate **1a** (3.28 g, 14 mmol) was dissolved in different solvents. The base (17 mmol) and 2-chloro-5-(chloromethyl)pyridine (2.27 g, 14 mmol) were then added. The mixture was stirred at room temperature overnight. Water was added, the mixture was extracted with 30 mL ethyl acetate three times, and the organic phase was washed once with saturated brine [32]. The solvent was removed in vacuo, and the residue was subjected to column chromatography [petroleum ether/ethyl acetate (*v*/*v* = 4:1)] to obtain target compounds **3a** and [petroleum ether/ethyl acetate (*v*/*v* = 1:2)] to obtain target compounds **2a**.

**2b**~**2k** and **3b**~**3k** were obtained at room temperature, in the presence of K_2_CO_3_ as the base in DMF solution.

#### 3.1.3. Chemical Properties of the Compounds

*(E)-N*-(1-((6-chloropyridin-3-yl)methyl)pyridin-2(1*H*)-ylidene)benzenesulfonamide (**2a**): white solid, Yield 69.0%. m.p. 169.8~170.3 °C; ^1^H NMR (500 MHz, Chloroform-*d*) *δ* 8.27 (d, *J* = 2.5 Hz, 1H), 7.83–7.77 (m, 2H), 7.74 (d, *J* = 9.1 Hz, 1H), 7.66 (dd, *J* = 6.8, 1.7 Hz, 1H), 7.58 (dd, *J* = 8.3, 2.6 Hz, 1H), 7.56–7.46 (m, 2H), 7.42 (t, *J* = 7.5 Hz, 2H), 7.18 (d, *J* = 8.2 Hz, 1H), 6.58 (td, *J* = 6.8, 1.4 Hz, 1H), 5.28 (s, 2H); ^13^C NMR (126 MHz, Chloroform-*d*) *δ* 155.3, 151.5, 149.4, 140.7, 139.2, 138.7, 131.6, 129.6, 128.7, 126.1, 124.4, 118.5, 111.5, 53.2; HR-MS (m/z): Calcd. for C_17_H_14_ClN_3_NaO_2_S (M+Na)^+^, 382.0393; found, 382.0394.

*(E)-N*-(1-((6-chloropyridin-3-yl)methyl)pyridin-2(1*H*)-ylidene)-2-methylbenzenesulfonamide (**2b**): white solid, Yield 47.9%. m.p. 150.7~151.4 °C; ^1^H NMR (500 MHz, Chloroform-*d*) *δ* 8.23 (d, *J* = 2.6 Hz, 1H), 8.01 (dd, *J* = 7.8, 1.4 Hz, 1H), 7.70–7.63 (m, 2H), 7.56–7.47 (m, 2H), 7.38 (td, *J* = 7.4, 1.3 Hz, 1H), 7.28 (s, 1H), 7.21 (d, *J* = 7.5 Hz, 1H), 7.15 (d, *J* = 8.2 Hz, 1H), 6.55 (td, *J* = 6.8, 1.2 Hz, 1H), 5.28 (s, 2H), 2.45 (s, 3H); ^13^C NMR (126 MHz, Chloroform-*d*) *δ* 155.4, 149.2, 141.2, 140.5, 139.1, 138.7, 137.0, 132.1, 131.7, 129.7, 127.7, 125.7, 124.4, 118.7, 111.3, 53.1, 20.4; HR-MS (m/z): Calcd. for C_18_H_16_ClN_3_NaO_2_S (M+Na)^+^, 396.0550; found, 396.0556.

*(E)-N*-(1-((6-chloropyridin-3-yl)methyl)pyridin-2(1*H*)-ylidene)-3-methylbenzenesulfonamide (**2c**): white solid, Yield 38.3%. m.p. 120.5~120.6 °C; ^1^H NMR (500 MHz, Chloroform-*d*) *δ* 8.29 (d, *J* = 2.2 Hz, 1H), 7.75 (d, *J* = 5.9 Hz, 1H), 7.67 (d, *J* = 9.2 Hz, 1H), 7.59 (dt, *J* = 8.2, 4.3 Hz, 2H), 7.54 (s, 1H), 7.53–7.49 (m, 1H), 7.28 (d, *J* = 6.7 Hz, 2H), 7.17 (d, *J* = 8.2 Hz, 1H), 6.57 (t, *J* = 6.5 Hz, 1H), 5.29 (s, 2H), 2.35 (s, 3H); ^13^C NMR (126 MHz, Chloroform-*d*) *δ* 155.3, 151.4, 149.5, 143.0, 140.7, 139.3, 139.0, 138.8, 132.4, 129.7, 128.5, 126.6, 124.3, 123.2, 118.3, 111.5, 53.3, 21.4; HR-MS (m/z): Calcd. for C_18_H_16_ClN_3_NaO_2_S (M+Na)^+^, 396.0550; found, 396.0559.

*(E)-N*-(1-((6-chloropyridin-3-yl)methyl)pyridin-2(1*H*)-ylidene)-4-methylbenzenesulfonamide (**2d**): light yellow solid, Yield 62.0%. m.p. 185.8~186.8 °C; ^1^H NMR (500 MHz, Chloroform-*d*) *δ* 8.55–8.42 (m, 2H), 7.91–7.76 (m, 2H), 7.60 (dd, *J* = 17.7, 8.2 Hz, 3H), 7.53 (d, *J* = 8.9 Hz, 1H), 7.33 (d, *J* = 8.0 Hz, 2H), 6.90 (t, *J* = 7.4 Hz, 1H), 5.48 (s, 2H), 2.42 (s, 3H); ^13^C NMR (126 MHz, DMSO-*d6*) *δ* 155.0, 150.5, 150.1, 142.3, 141.9, 141.6, 141.0, 140.0, 131.5, 129.6, 126.3, 124.6, 117.0, 112.1, 53.0, 21.4; HR-MS (m/z): Calcd. for C_18_H_16_ClN_3_NaO_2_S (M+Na)^+^, 396.0550; found, 396.0548.

(*E*)-2-chloro-*N*-(1-((6-chloropyridin-3-yl)methyl)pyridin-2(1*H*)-ylidene)benzenesulfonamide (**2e**): light yellow solid, Yield 52.1%. m.p. 148.3~149.3 °C; ^1^H NMR (500 MHz, Chloroform-*d*) *δ* 8.21 (d, *J* = 2.5 Hz, 1H), 8.13 (dd, *J* = 8.0, 1.8 Hz, 1H), 7.77 (dd, *J* = 17.9, 1.5 Hz, 2H), 7.59–7.51 (m, 2H), 7.44–7.39 (m, 1H), 7.39–7.33 (m, 2H), 7.07 (d, *J* = 8.2 Hz, 1H), 6.62 (td, *J* = 6.8, 1.4 Hz, 1H), 5.30 (s, 2H); ^13^C NMR (126 MHz, Chloroform-*d*) *δ* 155.4, 151.3, 149.4, 140.7, 140.7, 139.2, 138.9, 132.5, 132.0, 131.5, 129.6, 129.5, 126.7, 124.2, 118.8, 111.8, 53.4; HR-MS (m/z): Calcd. for C_17_H_13_Cl_2_N_3_NaO_2_S (M+Na)^+^, 416.0004; found, 416.0004.

(*E*)-3-chloro-N-(1-((6-chloropyridin-3-yl)methyl)pyridin-2(1*H*)-ylidene)benzenesulfonamide (**2f**): white solid, Yield 46.1%. m.p. 155.7~165.2 °C; ^1^H NMR (500 MHz, Chloroform-*d*) *δ* 8.28 (d, *J* = 2.5 Hz, 1H), 7.77–7.68 (m, 3H), 7.68–7.63 (m, 1H), 7.62–7.54 (m, 2H), 7.44 (dt, *J* = 8.0, 1.4 Hz, 1H), 7.35 (t, *J* = 7.9 Hz, 1H), 7.23 (d, *J* = 8.3 Hz, 1H), 6.65 (td, *J* = 6.8, 1.4 Hz, 1H), 5.30 (s, 2H); ^13^C NMR (126 MHz, Chloroform-*d*) *δ* 155.3, 149.3, 144.9, 141.1, 139.1, 138.9, 134.7, 131.7, 130.0, 129.4, 126.3, 124.5, 124.3, 1184, 111.9, 53.5; HR-MS (m/z): Calcd. for C_17_H_13_Cl_2_N_3_NaO_2_S (M+Na)^+^, 416.0004; found, 416.0003.

(*E*)-4-chloro-*N*-(1-((6-chloropyridin-3-yl)methyl)pyridin-2(1*H*)-ylidene)benzenesulfonamide (**2g**): white solid, Yield 71.3%. m.p. 153.6~154.2 °C; ^1^H NMR (500 MHz, Chloroform-*d*) *δ* 8.28 (d, *J* = 2.4 Hz, 1H), 7.73 (d, *J* = 6.7 Hz, 3H), 7.67 (d, *J* = 7.8 Hz, 1H), 7.59–7.52 (m, 2H), 7.40–7.35 (m, 2H), 7.22 (d, *J* = 8.2 Hz, 1H), 6.61 (td, *J* = 6.8, 1.3 Hz, 1H), 5.28 (s, 2H); ^13^C NMR (126 MHz, Chloroform-*d*) *δ* 155.3, 151.7, 149.3, 141.8, 140.9, 139.0, 138.6, 137.9, 129.5, 128.9, 127.7, 124.5, 118.6, 111.7, 53.4; HR-MS (m/z): Calcd. for C_17_H_13_Cl_2_N_3_NaO_2_S (M+Na)^+^, 416.0004; found, 416.0004.

(*E*)-3-bromo-*N*-(1-((6-chloropyridin-3-yl)methyl)pyridin-2(1*H*)-ylidene)benzenesulfonamide (**2h**): white solid, Yield 60.6%. m.p. 161.6~162.3 °C; ^1^H NMR (500 MHz, Chloroform-*d*) *δ* 8.27 (d, *J* = 2.3 Hz, 1H), 7.91 (t, *J* = 1.6 Hz, 1H), 7.72 (d, *J* = 7.5 Hz, 3H), 7.62–7.55 (m, 3H), 7.32–7.27 (m, 1H), 7.24 (d, *J* = 8.2 Hz, 1H), 6.72–6.60 (m, 1H), 5.30 (s, 2H); ^13^C NMR (126 MHz, Chloroform-*d*) *δ* 154.3, 150.7, 148.2, 144.1, 134.0, 138.1, 137.6, 133.6, 129.2, 128.3, 128.2, 123.7, 123.5, 121.6, 117.6, 110.8, 52.5; HR-MS (m/z): Calcd. for C_17_H_14_BrClN_3_O_2_S (M+H)^+^, 437.9678; found, 437.9674.

*(E)-N*-(1-((6-chloropyridin-3-yl)methyl)pyridin-2(1*H*)-ylidene)-4-methoxybenzenesulfonamide (**2i**): white solid, Yield 21.3%. m.p. 146.2~146.6 °C; ^1^H NMR (500 MHz, DMSO-*d*6) *δ* 8.27 (d, *J* = 2.3 Hz, 1H), 7.79–7.70 (m, 3H), 7.64–7.56 (m, 2H), 7.51 (ddd, *J* = 9.0, 6.9, 1.7 Hz, 1H), 7.21 (d, *J* = 8.2 Hz, 1H), 6.95–6.84 (m, 2H), 6.55 (td, *J* = 6.8, 1.2 Hz, 1H), 5.27 (s, 2H), 3.85 (s, 3H); ^13^C NMR (126 MHz, Chloroform-*d*) *δ* 162.0, 155.3, 151.6, 149.3, 140.5, 139.3, 138.4, 135.3, 129.7, 128.2, 124.5, 118.6, 113.7, 111.1, 55.5, 53.1; HR-MS (m/z): Calcd. for C_18_H_17_ClN_3_O_3_S (M+H)^+^, 390.0679; found, 390.0674.

*(E)-N*-(1-((6-chloropyridin-3-yl)methyl)pyridin-2(1*H*)-ylidene)-4-nitrobenzenesulfonamide (**2j**): white solid, Yield 51.5%. m.p. 173.1~173.6 °C; ^1^H NMR (500 MHz, Chloroform-*d*) *δ* 8.48–8.36 (m, 1H), 8.27 (d, *J* = 2.3 Hz, 1H), 8.24–8.16 (m, 2H), 7.83 (t, *J* = 7.7 Hz, 3H), 7.62 (dd, *J* = 8.3, 2.5 Hz, 1H), 7.51 (d, *J* = 9.0 Hz, 1H), 7.41 (d, *J* = 8.2 Hz, 1H), 6.89 (td, *J* = 6.9, 1.1 Hz, 1H), 5.36 (s, 2H); ^13^C NMR (126 MHz, DMSO-*d6*) *δ* 154.8, 150.2, 150.1, 149.5, 149.3, 143.1, 142.0, 139.7, 131.4, 127.5, 124.6, 124.6, 117.1, 113.2, 53.5; HR-MS (m/z): Calcd. for C_17_H_14_ClN_4_O_4_S (M+Na)^+^, 405.0424; found, 405.0421.

(*E*)-2,4-dichloro-*N*-(1-((6-chloropyridin-3-yl)methyl)pyridin-2(1*H*)-ylidene)benzenesulfonamide (**2k**): light yellow solid, Yield 7.3%. m.p. 208.2~208.6 °C; ^1^H NMR (500 MHz, Chloroform-*d*) *δ* 8.22 (d, *J* = 2.3 Hz, 1H), 8.10 (d, *J* = 8.5 Hz, 1H), 7.86 (d, *J* = 9.1 Hz, 1H), 7.69–7.63 (m, 1H), 7.63–7.54 (m, 2H), 7.39 (d, *J* = 2.0 Hz, 1H), 7.35 (dd, *J* = 8.5, 2.0 Hz, 1H), 7.15 (d, *J* = 8.2 Hz, 1H), 6.65 (td, *J* = 6.8, 1.2 Hz, 1H), 5.28 (s, 2H); ^13^C NMR (126 MHz, Chloroform-*d*) *δ* 155.5, 151.7, 149.3, 140.7, 139.4, 139.0, 138.4, 138.1, 133.0, 131.2, 130.6, 129.4, 127.0, 124.3, 119.2, 111.9, 53.5; HR-MS (m/z): Calcd. for C_17_H_13_Cl_3_N_3_O_2_S (M+H)^+^, 427.9794; found, 427.9797.

*N*-((6-chloropyridin-3-yl)methyl)-*N*-(pyridin-2-yl)benzenesulfonamide (**3a**): white solid, Yield 15.4%. m.p. 89.9~91.3 °C; ^1^H NMR (500 MHz, Chloroform-*d*) *δ* 8.30–8.23 (m, 2H), 8.02 (s, 1H), 7.73 (dd, *J* = 8.2, 2.5 Hz, 1H), 7.67–7.64 (m, 1H), 7.62–7.55 (m, 4H), 7.50–7.43 (m, 2H), 7.21 (dd, *J* = 8.2, 0.7 Hz, 1H), 7.10 (ddd, *J* = 7.4, 4.9, 1.1 Hz, 1H), 4.99 (s, 2H); ^13^C NMR (126 MHz, Chloroform-*d*) *δ* 151.3, 150.6, 149.9, 148.1, 139.2, 138.0, 133.3, 131.3, 129.1, 127.4, 124.1, 122.5, 122.0, 47.9; HR-MS (m/z): Calcd. for C_17_H_15_ClN_3_O_2_S (M+H)^+^, 382.0393; found, 382.0398.

*N*-((6-chloropyridin-3-yl)methyl)-2-methyl-*N*-(pyridin-2-yl)benzenesulfonamide (**3b**): yellow liquid, Yield 12.3%; ^1^H NMR (500 MHz, Chloroform-*d*) *δ* 8.34–8.27 (m, 2H), 7.95 (d, *J* = 9.1 Hz, 1H), 7.76 (dd, *J* = 8.2, 2.5 Hz, 1H), 7.63–7.55 (m, 1H), 7.50 (d, *J* = 8.3 Hz, 1H), 7.45 (t, *J* = 8.1 Hz, 1H), 7.30 (t, *J* = 7.4 Hz, 1H), 7.26 (d, *J* = 7.5 Hz, 1H), 7.21 (d, *J* = 8.2 Hz, 1H), 7.04 (dd, *J* = 6.8, 5.3 Hz, 1H), 5.12 (s, 2H), 2.33 (s, 3H); ^13^C NMR (126 MHz, Chloroform-*d*) *δ* 151.2, 150.6, 149.8, 148.1, 139.09, 137.9, 137.1, 133.2, 133.0, 131.7, 129.9, 126.2, 124.1, 121.4, 121.1, 47.7, 20.3; HR-MS (m/z): Calcd. for C_18_H_17_ClN_3_O_2_S (M+H)^+^, 396.0550; found, 396.0550.

*N*-((6-chloropyridin-3-yl)methyl)-3-methyl-*N*-(pyridin-2-yl)benzenesulfonamide (**3c**): white solid, Yield 24.3%. m.p. 92.3~93.4 °C; ^1^H NMR (500 MHz, Chloroform-*d*) *δ* 8.31–8.22 (m, 2H), 7.76–7.71 (m, 1H), 7.66 (td, *J* = 7.8, 2.0 Hz, 1H), 7.58 (d, *J* = 8.1 Hz, 1H), 7.40–7.35 (m, 3H), 7.35–7.30 (m, 1H), 7.20 (d, *J* = 8.2 Hz, 1H), 7.14–7.04 (m, 1H), 4.99 (s, 2H), 2.36 (s, 3H); ^13^C NMR (126 MHz, Chloroform-*d*) *δ* 151.4, 150.6, 149.9, 148.0, 139.4, 139.2, 138.0, 137.9, 134.1, 131.4, 129.0, 127.8, 124.5, 124.1, 122.4, 121.8, 47.9, 21.3; HR-MS (m/z): Calcd. for C_18_H_17_ClN_3_O_2_S (M+H)^+^, 396.0550; found, 396.0550.

*N*-((6-chloropyridin-3-yl)methyl)-4-methyl-*N*-(pyridin-2-yl)benzenesulfonamide (**3d**): light yellow solid, Yield 10.7%. m.p. 81.3~82.4 °C; ^1^H NMR (500 MHz, Chloroform-*d*) *δ* 8.31–8.21 (m, 2H), 7.72 (dd, *J* = 8.2, 2.5 Hz, 1H), 7.64 (ddd, *J* = 8.1, 7.3, 2.0 Hz, 1H), 7.57 (dt, *J* = 8.2, 1.1 Hz, 1H), 7.50–7.44 (m, 2H), 7.25 (d, *J* = 8.0 Hz, 2H), 7.19 (d, *J* = 8.2 Hz, 1H), 7.08 (ddd, *J* = 7.3, 4.8, 1.1 Hz, 1H), 4.97 (s, 2H), 2.41 (s, 3H; ^13^C NMR (126 MHz, Chloroform-*d*) *δ* 151.4, 150.5, 149.9, 148.0, 144.2, 139.2, 138.0, 135.0, 131.4, 129.8, 127.4, 124.0, 122.5, 121.8, 47.8, 21.6; HR-MS (m/z): Calcd. for C_18_H_17_ClN_3_O_2_S (M+H)^+^, 396.0550; found, 396.0554.

2-chloro-*N*-((6-chloropyridin-3-yl)methyl)-*N*-(pyridin-2-yl)benzenesulfonamide (**3e**): light yellow solid, Yield 13.8%. m.p. 62.8~64.0 °C; ^1^H NMR (500 MHz, Chloroform-*d*) *δ* 8.35 (d, *J* = 2.4 Hz, 1H), 8.31–8.24 (m, 1H), 8.14–8.07 (m, 1H), 7.78 (dd, *J* = 8.3, 2.5 Hz, 1H), 7.57 (ddd, *J* = 9.3, 7.4, 2.0 Hz, 1H), 7.52–7.43 (m, 3H), 7.39 (ddd, *J* = 8.5, 6.7, 2.1 Hz, 1H), 7.22 (d, *J* = 8.2 Hz, 1H), 7.02 (ddd, *J* = 7.3, 4.9, 1.0 Hz, 1H), 5.28 (s, 2H); ^13^C NMR (126 MHz, Chloroform-*d*) *δ* 150.9, 150.6, 149.8, 148.1, 139.0, 137.9, 136.6, 134.2, 132.3, 132.3, 131.8, 127.0, 124.2, 121.2, 119.8, 48.2; HR-MS (m/z): Calcd. for C_17_H_14_Cl_2_N_3_O_2_S (M+H)^+^, 416.0004; found, 416.0006.

3-chloro-*N*-((6-chloropyridin-3-yl)methyl)-*N*-(pyridin-2-yl)benzenesulfonamide (**3f**): white solid, Yield 11.8%. m.p. 102.3~103.3 °C; ^1^H NMR (500 MHz, Chloroform-*d*) *δ* 8.29 (dd, *J* = 4.9, 1.9 Hz, 1H), 8.27 (d, *J* = 2.4 Hz, 1H), 7.74–7.65 (m, 2H), 7.60–7.50 (m, 3H), 7.45 (dt, *J* = 7.9, 1.5 Hz, 1H), 7.40 (d, *J* = 15.7 Hz, 1H), 7.21 (d, *J* = 8.2 Hz, 1H), 7.14 (ddd, *J* = 7.5, 4.9, 1.0 Hz, 1H), 4.98 (s, 2H); ^13^C NMR (126 MHz, Chloroform-*d*) *δ* 151.0, 150.8, 149.9, 148.3, 139.6, 139.2, 138.2, 135.3, 133.4, 130.9, 130.4, 127.6, 125.5, 124.1, 122.83, 122.4, 48.2; HR-MS (m/z): Calcd. for C_17_H_14_Cl_2_N_3_O_2_S (M+H)^+^, 416.0004; found, 416.0002.

4-chloro-*N*-((6-chloropyridin-3-yl)methyl)-*N*-(pyridin-2-yl)benzenesulfonamide (**3g**): white solid, Yield 9.4%. m.p. 83.6~84.0 °C; ^1^H NMR (500 MHz, Chloroform-*d*) *δ* 8.27 (dd, *J* = 8.7, 2.1 Hz, 2H), 7.71 (dd, *J* = 8.2, 2.5 Hz, 1H), 7.69–7.62 (m, 1H), 7.56–7.49 (m, 3H), 7.46–7.40 (m, 2H), 7.21 (d, *J* = 8.2 Hz, 1H), 7.15–7.08 (m, 1H), 4.96 (s, 2H); ^13^C NMR (126 MHz, Chloroform-*d*) *δ* 151.1, 150.8, 149.9, 148.3, 139.9, 139.2, 138.2, 136.4, 131.0, 129.4, 128.9, 124.1, 122.9, 122.3, 48.1; HR-MS (m/z): Calcd. for C_17_H_14_Cl_2_N_3_O_2_S (M+H)^+^, 416.0004; found, 416.0006.

3-bromo-*N*-((6-chloropyridin-3-yl)methyl)-*N*-(pyridin-2-yl)benzenesulfonamide (**3h**): light yellow solid, Yield 2.7%. m.p. 125.2~125.5 °C; ^1^H NMR (500 MHz, Chloroform-*d*) *δ* 8.31–8.27 (m, 1H), 8.26 (d, *J* = 2.1 Hz, 1H), 7.74–7.70 (m, 3H), 7.67 (dd, *J* = 7.5, 1.9 Hz, 1H), 7.53 (d, *J* = 8.1 Hz, 1H), 7.49 (dt, *J* = 7.9, 1.1 Hz, 1H), 7.33 (t, *J* = 8.0 Hz, 1H), 7.22 (d, *J* = 8.2 Hz, 1H), 7.14 (ddd, *J* = 7.4, 4.8, 0.9 Hz, 1H), 4.97 (s, 2H); ^13^C NMR (126 MHz, Chloroform-*d*) *δ* 151.0, 150.8, 149.9, 148.3, 139.7, 139.2, 138.2, 136.2, 130.9, 130.6, 130.4, 125.9, 124.2, 123.1, 122.9, 122.4, 48.2; HR-MS (m/z): Calcd. for C_17_H_14_BrClN_3_O_2_S (M+H)^+^, 437.9671; found, 437.9678.

*N*-((6-chloropyridin-3-yl)methyl)-4-methoxy-*N*-(pyridin-2-yl)benzenesulfonamide (**3i**): white solid, Yield 20.5%. m.p. 84.0~85.0 °C; ^1^H NMR (500 MHz, Chloroform-*d*) *δ* 8.31–8.22 (m, 2H), 7.72 (dd, *J* = 8.2, 2.5 Hz, 1H), 7.67–7.61 (m, 1H), 7.57 (d, *J* = 8.2 Hz, 1H), 7.54–7.48 (m, 2H), 7.20 (d, *J* = 8.2 Hz, 1H), 7.09 (ddd, *J* = 7.3, 4.9, 0.9 Hz, 1H), 6.96–6.88 (m, 2H), 4.96 (s, 2H), 3.85 (s, 3H); ^13^C NMR (126 MHz, Chloroform-*d*) *δ* 163.3, 151.5, 150.5, 149.9, 148.0, 139.2, 138.0, 131.5, 129.6, 129.5, 124.1, 122.6, 121.8, 114.3, 55.6, 47.8; HR-MS (m/z): Calcd. for C_18_H_17_ClN_3_O_3_S (M+H)^+^, 390.0679; found, 390.0675.

*N*-((6-chloropyridin-3-yl)methyl)-4-nitro-*N*-(pyridin-2-yl)benzenesulfonamide (**3j**): white solid, Yield 9.4%. m.p. 120.0~120.8 °C; ^1^H NMR (500 MHz, Chloroform-*d*) *δ* 8.31–8.22 (m, 2H), 7.72 (dd, *J* = 8.2, 2.5 Hz, 1H), 7.67–7.61 (m, 1H), 7.57 (d, *J* = 8.2 Hz, 1H), 7.54–7.48 (m, 2H), 7.20 (d, *J* = 8.2 Hz, 1H), 7.09 (ddd, *J* = 7.3, 4.9, 0.9 Hz, 1H), 6.96–6.88 (m, 2H), 4.96 (s, 2H), 3.85 (s, 3H); ^13^C NMR (126 MHz, Chloroform-*d*) *δ* 151.1, 150.7, 150.4, 149.9, 148.6, 143.6, 139.2, 138.5, 130.4, 128.8, 124.3, 124.3, 123.4, 122.9; HR-MS (m/z): Calcd. for C_17_H_14_ClN_4_O_4_S (M+H)^+^, 405.0424; found, 405.0420.

2,4-dichloro-*N*-((6-chloropyridin-3-yl)methyl)-*N*-(pyridin-2-yl)benzenesulfonamide (**3k**): white solid, Yield 14.7%. m.p. 126.1~126.5 °C; ^1^H NMR (500 MHz, Chloroform-*d*) *δ* 8.33 (d, *J* = 2.2 Hz, 1H), 8.31–8.25 (m, 1H), 8.02 (d, *J* = 8.6 Hz, 1H), 7.76 (dd, *J* = 8.2, 2.4 Hz, 1H), 7.59 (td, *J* = 7.9, 1.8 Hz, 1H), 7.48 (d, *J* = 1.8 Hz, 1H), 7.42 (d, *J* = 8.2 Hz, 1H), 7.36 (dd, *J* = 8.6, 1.9 Hz, 1H), 7.23 (d, *J* = 8.2 Hz, 1H), 7.06 (dd, *J* = 7.3, 4.9 Hz, 1H), 5.25 (s, 2H); ^13^C NMR (126 MHz, Chloroform-*d*) *δ* 150.8, 150.7, 149.8, 148.3, 140.1, 139.0, 138.1, 135.2, 133.3, 133.2, 132.0, 131.5, 127.4, 124.2, 121.6, 120.2, 48.4; HR-MS (m/z): Calcd. for C_17_H_13_Cl_3_N_3_O_2_S (M+H)^+^, 427.9794; found, 427.9795.

### 3.2. Insecticidal Activity Test

Insecticidal activity was tested using the leaf-dip method [28]. The test insects were *A. glycines*, a normal population raised in the laboratory. The target compounds were dissolved in dimethyl sulfoxide and diluted with distilled water containing 3‰ Tween-80 buffers to the desired concentration. Soybean leaves were cut into disks (20 mm in diameter), dipped into the solutions for 15 s, taken out and the excess solutions absorbed using filter paper. The soybean leaves were then placed in a twelve-well cell plate with 1 mL of agar solution (2%) at the bottom of each well. Approximately 20 wingless aphids were placed on the leaves and sealed with rice paper and placed in an incubator at 25 ± 2 °C. This was repeated 3 times for each compound and the results were checked for pest mortality at 48 h. **PYM** and **FLP** were positive controls. LC_50_ values were calculated by SPSS v.23.0.

### 3.3. Oral and Contact Toxicity to Apis mellifera

*A. mellifera* worker bees were collected from a healthy colony kept in the laboratory at the campus of Guangxi University, China. All the test bees were reared in the incubator at 25 ± 2 °C and a relative humidity of 50–70%, in darkness.

The compounds were first dissolved in acetone to obtain a high concentration solution. In these experiments, three replicate groups were set up for each treatment and each replicate contained 10 bees. Complete blank control (sucrose water) and solvent control were carried out simultaneously under the same conditions. The compounds were exposed to the bees via oral and contact routes as described previously [34,35,36]. In brief, for the oral toxicity test, the above solution was diluted in 50% sucrose water to a certain concentration, and each bee was recorded to consume an average of 60 μL of sucrose water containing the active ingredient at 24 h. The bees were fed with the solution continuously for 72 h, and then their deaths were recorded. For the contact toxicity test, after the bees were anesthetized with carbon dioxide, 2 μL of the solution was dropped onto the mid-chest of each bee. When the solution evaporated, the bees were transferred to cages where they were fed with 50% sucrose water for 72 h, and then their deaths were recorded [34,35,36].

### 3.4. Molecular Docking

The PDB file for the complex crystals of insect nicotinic acetylcholine-binding protein and imidacloprid (PDB ID: 3C79) can be downloaded from the PDB library (http://www.rcsb.org accessed on 18 January 2021) [12]. The honeybee nicotinic acetylcholine-receptor protein was constructed by homology in the laboratory [18]. The structure optimization of the compound, the preprocessing of the protein structure and the molecular docking operation were all completed using the Sybyl v7.3 software (Tripos, St. Louis, MI, USA). The mapping of small-molecule binding modes was performed in Pymol software.

### 3.5. Density Functional Theory (DFT) Analysis

The most active compound, **2j**, and lead compound, **FLP**, were selected and drawn in GaussView 6.0. The two structures were optimized using the DFT-B3LYP/6-311G method in the Gaussian 09W package. Vibration analysis indicated that the two optimized structures were for the minimum points in the potential energy surfaces. This indicated no virtual frequencies and proved that the optimized structures were stable. All the convergent precisions were the system default values.

## 4. Conclusions

In this study, 22 new compounds were designed and synthesized with two different isomers based on the commercial insecticide **FLP**. All target compounds were confirmed by ^1^H NMR, ^13^C NMR and HRMS spectra. The bioassay results showed that several target compounds exhibited considerable insecticidal activity against *A. glycines*. In particular, compound **2j** exhibited the best insecticidal activity (LC_50_ = 20.93 mg/L), and low acute oral and contact toxicity for *A. mellifera*, and is worthy of further modification. For compound **2a**~**2k** with high yields, the structure–activity relationships revealed that an electron-withdrawing group contributes more to the aphicidal activity than the electron-donating group. Meanwhile, the binding mode of compound **2j** with insect AChBP indicated that hydrogen bonding with residue Met116 could maintain a good affinity with insect AChBP, and exhibited a low bee-toxicity binding mode with honeybee nAChR. Therefore, compound **2j**, with its good insecticidal activity and low bee-toxicity, can be regarded as a lead compound for further optimization. Based on the present beneficial guidance results, further structural optimization is underway in our group.

## Figures and Tables

**Table 1:** Optimization of reaction of intermediate **1** with 2-chloro-5-(chloromethyl) pyridine.

Entry	Base	Solvent	2a Yield %	3a Yield %
1	K_2_CO_3_	MeOH	29.41	14.05
2	K_2_CO_3_	THF	32.68	16.99
3	K_2_CO_3_	MeCN	39.54	20.26
4	K_2_CO_3_	DMSO	49.35	11.44
5	K_2_CO_3_	DMF	68.95	15.36
6	Cs_2_CO_3_	DMF	65.95	16.01
7	Et_3_N	DMF	26.14	16.01
8	DIPEA	DMF	67.97	15.03
9	NaH	DMF	64.30	10.30
10	NaOH	DMF	65.36	0

Note: Et_3_N: triethylamine; DIPEA: N, N-diisopropylethylamine; K_2_CO_3_: potassium carbonate; Cs_2_CO_3_: caesium carbonate; NaOH: sodium hydroxide; NaH: sodium hydride; DMF: N, N-dimethylformamide; THF: tetrahydrofuran; MeCN: acetonitrile; DMSO: dimethyl sulfoxide; MeOH: methanol.

**Table 2:** Insecticidal activity against adults *A. glycines* by the target compounds (200 mg/L, 48 h).

Compd.	R	Mortality Rate ± St. D ^a^/%	Compd.	R	Mortality Rate ± St. D/%
**2a**	H	13.96 ± 1.82	**3a**	H	46.51 ± 7.91
**2b**	2-CH_3_	18.61 ± 4.80	**3b**	2-CH_3_	55.81 ± 3.63
**2c**	3-CH_3_	23.26 ± 3.14	**3c**	3-CH_3_	72.09 ± 3.14
**2d**	4-CH_3_	39.54 ± 3.63	**3d**	4-CH_3_	32.56 ± 4.80
**2e**	2-Cl	37.21 ± 11.33	**3e**	2-Cl	51.17 ± 3.14
**2f**	3-Cl	60.47 ± 6.54	**3f**	3-Cl	18.61 ± 3.63
**2g**	4-Cl	76.75 ± 4.80	**3g**	4-Cl	30.24 ± 6.29
**2h**	3-Br	68.15 ± 11.65	**3h**	3-Br	17.65 ± 5.77
**2i**	4-OCH_3_	49.02 ± 5.44	**3i**	4-OCH_3_	9.80 ± 2.72
**2j**	4-NO_2_	94.12 ± 2.89	**3j**	4-NO_2_	17.65 ± 9.43
**2k**	2,4-2Cl	57.50 ± 6.35	**3k**	2,4-2Cl	13.73 ± 2.72
PYM		81.62 ± 4.97	FLP		100

^a^: St. D = Standard deviation.

**Table 3:** LC_50_ values of target compounds **2g**, **2j** and **3c** against *A. glycines*.

Compd.	Y = ax + b	LC_50_ (mg/L)	95% CI ^a^	LC_90_ (mg/L)	r	χ^2^
**2g**	y = 1.25x − 2.26	63.74	51.85–80.51	666.88	0.96	5.68
**2j**	y = 1.85x − 2.44	20.93	16.18–26.26	336.51	0.98	2.26
**3c**	y = 1.85x − 3.71	102.38	87.35–123.14	463.35	0.97	6.31
PYM	y = 1.92x − 1.90	9.98	4.37–21.40	47.479	0.98	5.00
FLP	y = 1.99x + 1.47	0.19	0.12–0.29	0.934	0.95	4.47

^a^: Confidence interval.

**Table 4:** The results of bee toxicity experiments on *A. mellifera*.

Compd.	Mortality Rate % (Dose)		Bee-Toxicity ^b^
	Oral Toxicity	Contact Toxicity	
**2j**	3.33 (12 μg/bee)	13.33 (13.34 μg/bee)	Low
**FLP**	6.67 (60 μg/bee)	0 (30 μg/bee)	Low
**IMI** ^a^	2.82 × 10^−2^	5.78 × 10^−2^	High

Note ^a^: the LD_50_ values of **IMI**; ^b^: High toxic: LD_50_ < 2 μg/bee; Moderately toxic: 2.0 < LD_50_ ≤ 11μg/bee; Low-toxic: LD_50_ > 11 μg/bee [30].

## Data Availability

All data presented in this study are available in the article and in the Supplementary Material.

## Supplementary Materials

The following supporting information can be downloaded at: https://www.mdpi.com/article/10.3390/molecules27186133/s1, Tables S1–S3: Crystal structure data for compound **2a**; Figures S1–S67: ^1^H NMR, ^13^C NMR and HRMS of the compound.

## Sample Availability

Samples of the compounds **2a**~**2k** and **3a**~**3k** are available from the authors.

## References

[1] Dedryver C.A., le Ralec A., Fabre F. (2010). The Conflicting Relationships between Aphids and Men: A Review of Aphid Damage and Control Strategies. Comptes Rendus Biol..

[2] Giordanengo P., Brunissen L., Rusterucci C., Vincent C., van Bel A., Dinant S., Girousse C., Faucher M., Bonnemain J.L. (2010). Compatible Plant-Aphid Interactions: How Aphids Manipulate Plant Responses. Comptes Rendus Biol..

[3] Brault V., Uzest M., Monsion B., Jacquot E., Blanc S. (2010). Aphids as Transport Devices for Plant Viruses. Comptes Rendus Biol..

[4] Zeni V., Baliota G.V., Benelli G., Canale A., Athanassiou C.G. (2021). Diatomaceous Earth for Arthropod Pest Control: Back to the Future. Molecules.

[5] Matsuda K., Ihara M., Sattelle D.B. (2020). Neonicotinoid Insecticides: Molecular Targets, Resistance, and Toxicity. Annu. Rev. Pharmacol. Toxicol..

[6] Nauen R., Bielza P., Denholm I., Gorman K. (2008). Age-Specific Expression of Resistance to a Neonicotinoid Insecticide in the Whitefly Bemisia Tabaci. Pest Manag. Sci..

[7] Wang B., Cheng J., Xu Z., Xu X., Shao X., Li Z. (2012). Synthesis and Biological Activity Evaluation of Novel β-Substituted Nitromethylene Neonicotinoid Analogues. Molecules.

[8] Matsuda K., Buckingham S.D., Kleier D., Rauh J.J., Grauso M., Sattelle D.B., Buckingham S.D. (2001). Neonicotinoids: Insecticides Acting on Insect Nicotinic Acetylcholine Receptors. Trends Pharmacol. Sci..

[9] Brejc K., van Dijk W.J., Klaassen R.V., Schuurmans M., van der Oost J., Smit A.B., Sixma T.K. (2001). Crystal Structure of an ACh-Binding Protein Reveals the Ligand-Binding Domain of Nicotinic Receptors. Nature.

[10] Celie P.H.N., van Rossum-Fikkert S.E., van Dijk W.J., Brejc K., Smit A.B., Sixma T.K. (2004). Nicotine and Carbamylcholine Binding to Nicotinic Acetylcholine Receptors as Studied in AChBP Crystal Structures. Neuron.

[11] Ihara M., Okajima T., Yamashita A., Oda T., Hirata K., Nishiwaki H., Morimoto T., Akamatsu M., Ashikawa Y., Kuroda S. (2008). Crystal Structures of Lymnaea Stagnalis AChBP in Complex with Neonicotinoid Insecticides Imidacloprid and Clothianidin. Invertebr. Neurosci..

[12] Talley T.T., Harel M., Hibbs R.E., Radić Z., Tomizawa M., Casida J.E., Taylor P. (2008). Atomic Interactions of Neonicotinoid Agonists with AChBP: Molecular Recognition of the Distinctive Electronegative Pharmacophore. Proc. Natl. Acad. Sci. USA.

[13] Liu Z., Song R., Zhang D., Wu R., Liu T., Wu Z., Song B. (2022). New Synthetic Method and Insecticidal Activities of Novel Imidazopyridine Mesoionic Derivatives Containing an Ester Group. J. Agric. Food Chem..

[14] Tian P., Liu D., Liu Z., Shi J., He W., Qi P., Chen J., Song B. (2019). Design, Synthesis, and Insecticidal Activity Evaluation of Novel 4-(N, N-Diarylmethylamines)Furan-2(5H)-One Derivatives as Potential Acetylcholine Receptor Insecticides. Pest Manag. Sci..

[15] Mitchell E.A.D., Mulhauser B., Mulot M., Mutabazi A., Glauser G., Aebi A. (2017). A Worldwide Survey of Neonicotinoids in Honey. Science.

[16] Whitehorn P.R., O’Connor S., Wackers F.L., Goulson D. (2012). Neonicotinoid Pesticide Reduces Bumble Bee Colony Growth and Queen Production. Science.

[17] Chen Z., Yao X., Dong F., Duan H., Shao X., Chen X., Yang T., Wang G., Zheng Y. (2019). Ecological Toxicity Reduction of Dinotefuran to Honeybee: New Perspective from an Enantiomeric Level. Environ. Int..

[18] Xu X., Yang Z., Zhu K., Li H., Qin Z., Duan H. (2020). Computational Insight on the Binding and Selectivity of Target-Subunit-Dependent for Neonicotinoid Insecticides. J. Mol. Graph. Model..

[19] Holyoke C.W., Zhang W., Pahutski T.F., Lahm G.P., Tong M.H.T., Cordova D., Schroeder M.E., Benner E.A., Rauh J.J., Dietrich R.F. (2015). Triflumezopyrim: Discovery and Optimization of a Mesoionic Insecticide for Rice. ACS Symp. Ser..

[20] Onozaki Y., Horikoshi R., Ohno I., Kitsuda S., Durkin K.A., Suzuki T., Asahara C., Hiroki N., Komabashiri R., Shimizu R. (2017). Flupyrimin: A Novel Insecticide Acting at the Nicotinic Acetylcholine Receptors. J. Agric. Food Chem..

[21] Nauen R., Jeschke P., Velten R., Beck M.E., Ebbinghaus-Kintscher U., Thielert W., Wölfel K., Haas M., Kunz K., Raupach G. (2015). Flupyradifurone: A Brief Profile of a New Butenolide Insecticide. Pest Manag. Sci..

[22] Watson G.B., Siebert M.W., Wang N.X., Loso M.R., Sparks T.C. (2021). Sulfoxaflor—A Sulfoximine Insecticide: Review and Analysis of Mode of Action, Resistance and Cross-Resistance. Pestic. Biochem. Physiol..

[23] He W., Liu D., Gan X., Zhang J., Liu Z., Yi C., Song B. (2019). Synthesis and Biological Activity of Novel 1,3,4-Thiadiazolo[3,2-a]Pyrimidinone Mesoionic Derivatives. Chin. J. Org. Chem..

[24] Harmata M., Zheng P., Huang C., Gomes M.G., Ying W., Ranyanil K., Balan G., Calkins N.L., Chem J.A.P.A. (2007). Expedient Synthesis of Sulfinamides from Sulfonyl Chlorides Sulfinamides Were Synthesized from Sulfonyl Chlorides Using a Procedure Involving in Situ Reduction of Sulfonyl Chlorides. The Reaction Is Broad in Scope and Easy to Perform. Sulfinamides, Esp. J. Org. Chem..

[25] Cho S.Y., Fox E., McCully C., Bauch J., Marsh K., Balis F.M. (2007). Plasma and Cerebrospinal Fluid Pharmacokinetics of Intravenously Administered ABT-751 in Non-Human Primates. Cancer Chemother. Pharmacol..

[26] Kumar Parai M., Panda G., Srivastava K., Kumar Puri S. (2008). Design, Synthesis and Antimalarial Activity of Benzene and Isoquinoline Sulfonamide Derivatives. Bioorganic Med. Chem. Lett..

[27] Yang C., Li X., Wei J., Zhu F., Gang F., Wei S., Zhao Y., Zhang J., Wu W. (2018). Synthesis and Insecticidal Activity In Vitro and Vivo of Novel Benzenesulfonyl Derivatives Based on Potent Target Subunit H of V-ATPase. Bioorganic Med. Chem. Lett..

[28] Yang Z., Wu X., Zhang J., Lu X., Li X., Jiang Z., Song D., Duan H., Yang X. (2021). Screening and Optimization of Novel Low Bee-Toxicity Phenylacetohydrazone Compounds Based on Insect NAChR Selectivity. Chin. J. Org. Chem..

[29] Hung K.L.J., Kingston J.M., Albrecht M., Holway D.A., Kohn J.R. (2018). The Worldwide Importance of Honey Bees as Pollinators in Natural Habitats. Proc. R. Soc. B Biol. Sci..

[30] WSDA (2010). Pollinator Protection Requirements for Section 18 Emergency Exemptions and Section 24(c) Special Local Need Registration in Washington State.

[31] Zhang X., Xu H., Su H., Yang X., Sun T., Lu X., Shi F., Duan H., Liu X., Ling Y. (2022). Design, Synthesis, and Biological Activity of Novel Fungicides Containing a 1,2,3,4-Tetrahydroquinoline Scaffold and Acting as Laccase Inhibitors. J. Agric. Food Chem..

[32] Zhang N., Liu A., Ren Y., Zhou C., Cheng S., Xiang J., Liu X., Liu M., Huang M., Liu Z. (2018). Synthesis and Biological Activity of the Novel Insecticide: Flupyrimin. FINE Chem. Intermed..

[33] El-Zemity S.R., Badawy M.E., Khattab M.M., Marei A.E.-S. (2006). Structure and Acaridical Activity Relationship of Some Sulfonamide Derivatves against the Two-Spotted Spider Mite, Tetranychus Urticae (Koch). Int. J. Agric. Biol..

[34] Abdullah I., Gary S.R., Marla S. (2007). Field Trial of Honey Bee Colonies Bred for Mechanisms of Resistance against Varroa Destructor. Apidologie.

[35] Duke O.S., Powles B.S. (2008). Glyphosate: A Once-in-a-Century Herbicide. Pest Manag. Sci..

[36] Oruc H.H., Hranitz J.M., Sorucu A., Duell M., Cakmak I., Aydin L., Orman A. (2012). Determination of Acute Oral Toxicity of Flumethrin in Honey Bees. J. Econ. Entomol..

