# Supplementary material for: Design, Synthesis and Bioactivity of Novel Low Bee-Toxicity Compounds Based on Flupyrimin
Source: Molecules. 2022 Sep 19;27(18):6133. doi: 10.3390/molecules27186133 (PMC9505580; doi:10.3390/molecules27186133)
Supplement: Supplementary file 1 [file molecules-27-06133-s001.zip › molecules-1917871-supplementary.pdf]

Supporting Information

Design, Synthesis and Bioactivity of Novel Low Bee-Toxicity Compounds Based on Flupyrimin

Xingxing Lu [1], Huan Xu [1], Xiaoming Zhang [1], Tengda Sun [1], Yufan Lin [1], Yongheng Zhang [2], Honghong Li [2], Xuesheng Li [2], Xinling Yang [1,*], Hongxia Duan [1,*], Yun Ling [1,*]

[1] Innovation Center of Pesticide Research, Department of Applied Chemistry, College of Science, China Agricultural University, Beijing 100193, China
[2] Guangxi Key Laboratory of Agro-Environment and Agro-Product Safety, Agricultural College, Guangxi University, Nanning 530004, China

Table S1. Crystal data and structure refinement for compound **2a.**

Name	Parameter
CCDC No.	2071276
Empirical formula	$C_{17}H_{14}ClN_3O_2S$
Formula weight	359.82
Temperature / K	108.0(3)
Crystal system	monoclinic
Space group	P21/n
a / Å, b / Å, c / Å	7.1231(2), 11.6414(3), 19.1788(6)
α/°, β/°, γ/°	90.00, 90.262(4), 90.00
Volume / Å3	1590.34(9)
Z	4
ϱcalc / mg mm-3	1.503
μ / mm-1	0.387
F(000)	744
Crystal size / mm3	0.34 × 0.27 × 0.23
2Θ range for data collection	6.7 to 52°
Index ranges	-8 ≤ h ≤ 8, -14 ≤ k ≤ 9, -23 ≤ l ≤ 23
Reflections collected	7304
Independent reflections	3115[R(int) = 0.0269 (inf-0.9Å)]
Data/restraints/parameters	3115/0/217
Goodness-of-fit on F2	1.055
Final R indexes [I>2σ (I) i. e. Fo>4σ (Fo)]	R1 = 0.0378, wR2 = 0.0848
Final R indexes [all data]	R1 = 0.0456, wR2 = 0.0906
Largest diff. peak/hole / e Å-3	0.267/-0.458
Flack Parameters	N
Completeness	0.9971

Table S2. Bond lengths for compound **2a.**

Chemical bond	Lengths [Å]	Chemical bond	Lengths [Å]
S1-O2	1.4454(14)	C2-C3	1.379(3)
S1-O1	1.4514(14)	C2-C1	1.387(3)

S1-N1	1.5957(17)	C7-C8	1.355(3)
S1-C12	1.767(2)	C13-C14	1.379(3)
Cl1-C1	1.754(2)	C13-C12	1.393(3)
N2-C7	1.362(3)	C17-C12	1.390(3)
N2-C11	1.374(2)	C17-C16	1.390(3)
N2-C6	1.481(2)	C3-C4	1.390(3)
N3-C1	1.314(3)	C6-C4	1.510(3)
N3-C5	1.349(3)	C4-C5	1.388(3)
N1-C11	1.335(3)	C9-C8	1.405(3)
C10-C11	1.426(3)	C14-C15	1.387(3)
C10-C9	1.361(3)	C15-C16	1.382(3)

Table S3. Bond angles for compound **2a.**

Chemical bond	Angles [°]	Chemical bond	Angles [°]
O2-S1-O1	114.72(8)	C16-C17-C12	118.9(2)
O2-S1-N1	112.64(9)	C2-C3-C4	119.82(19)
O2-S1-C12	108.05(9)	N3-C1-Cl1	115.53(15)
O1-S1-N1	113.10(9)	N3-C1-C2	126.00(19)
O1-S1-C12	106.68(9)	C2-C1-Cl1	118.48(16)
N1-S1-C12	100.30(9)	N2-C6-C4	112.65(16)
C7-N2-C11	122.32(17)	C3-C4-C6	121.91(18)
C7-N2-C6	118.55(16)	C5-C4-C3	117.82(18)
C11-N2-C6	119.09(16)	C5-C4-C6	120.20(18)
C1-N3-C5	116.04(18)	C10-C9-C8	120.44(19)
C11-N1-S1	121.57(14)	C13-C14-C15	120.1(2)
C9-C10-C11	121.03(19)	N3-C5-C4	123.58(19)
C3-C2-C1	116.73(19)	C16-C15-C14	120.1(2)
C8-C7-N2	121.67(19)	C7-C8-C9	118.25(19)
N2-C11-C10	116.23(17)	C13-C12-S1	118.66(15)
N1-C11-N2	115.13(16)	C17-C12-S1	120.50(15)
N1-C11-C10	128.64(18)	C17-C12-C13	120.61(19)
C14-C13-C12	119.75(19)	C15-C16-C17	120.6(2)

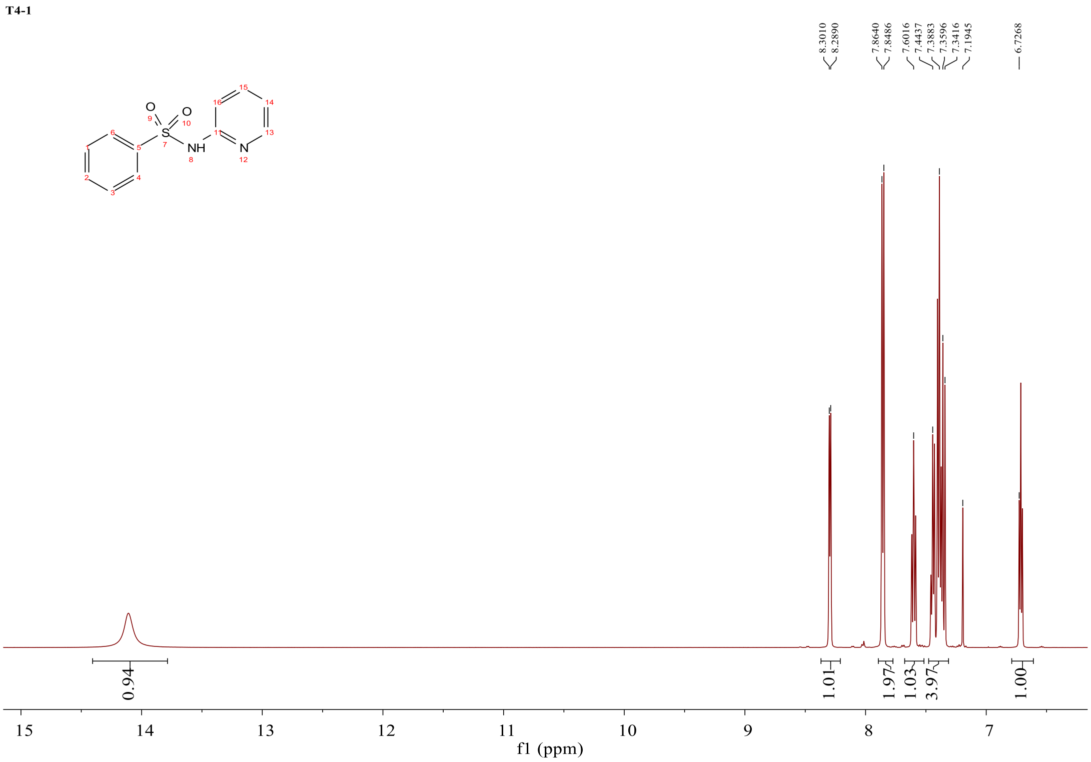

Figure S1. ^{1}H NMR of Intermediate **1.**

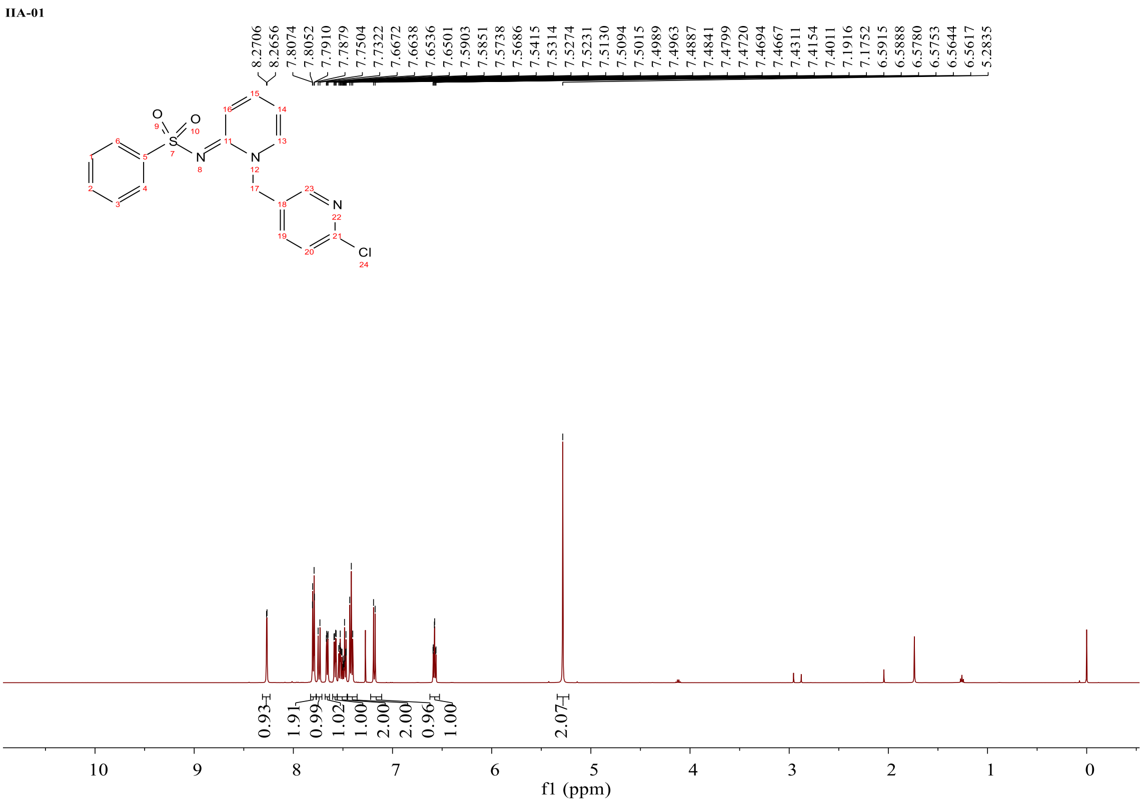

Figure S2. ^{1}H NMR of compound **2a.**

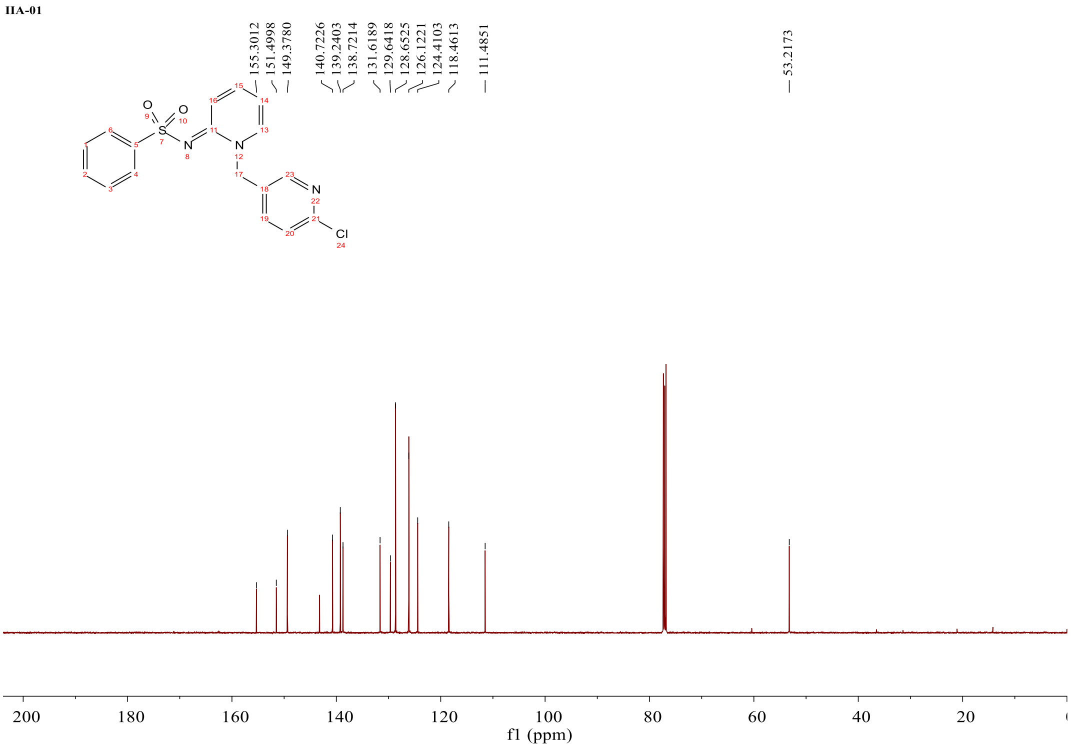

Figure S3. ^{13}C NMR of compound **2a.**

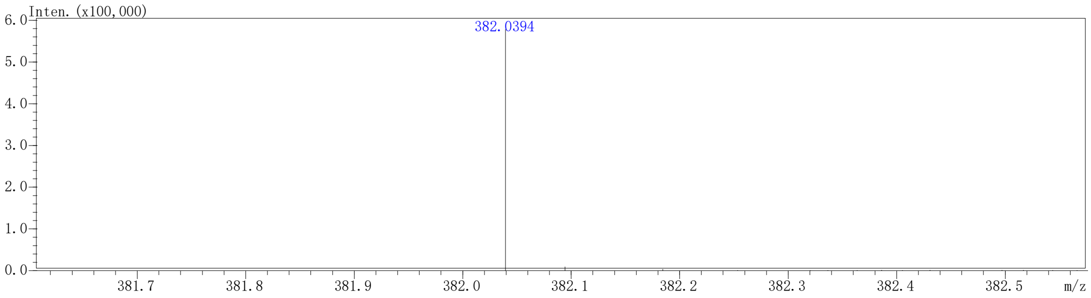

Figure S4. HRMS of compound **2a.**

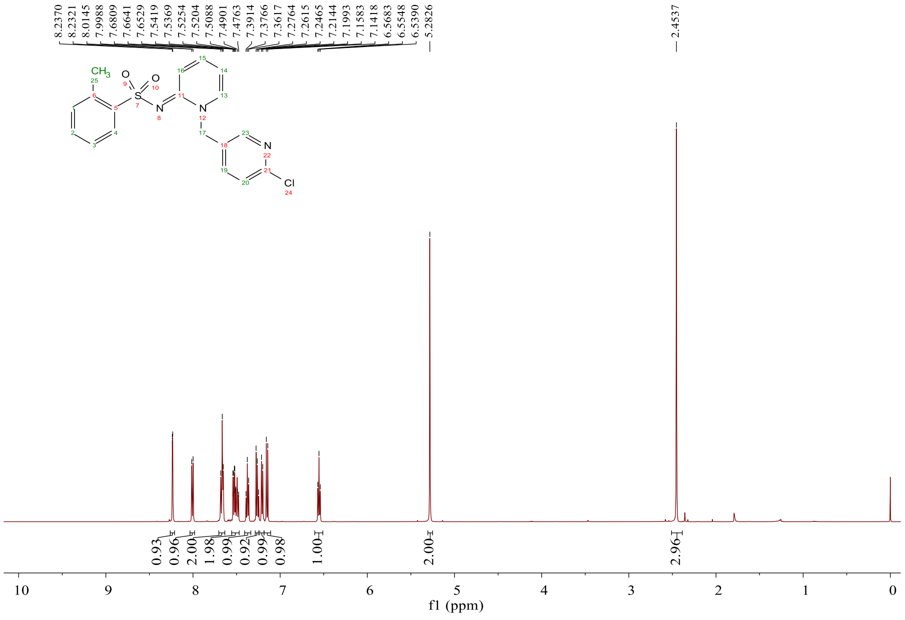

Figure S5. 1H NMR of compound 2b.

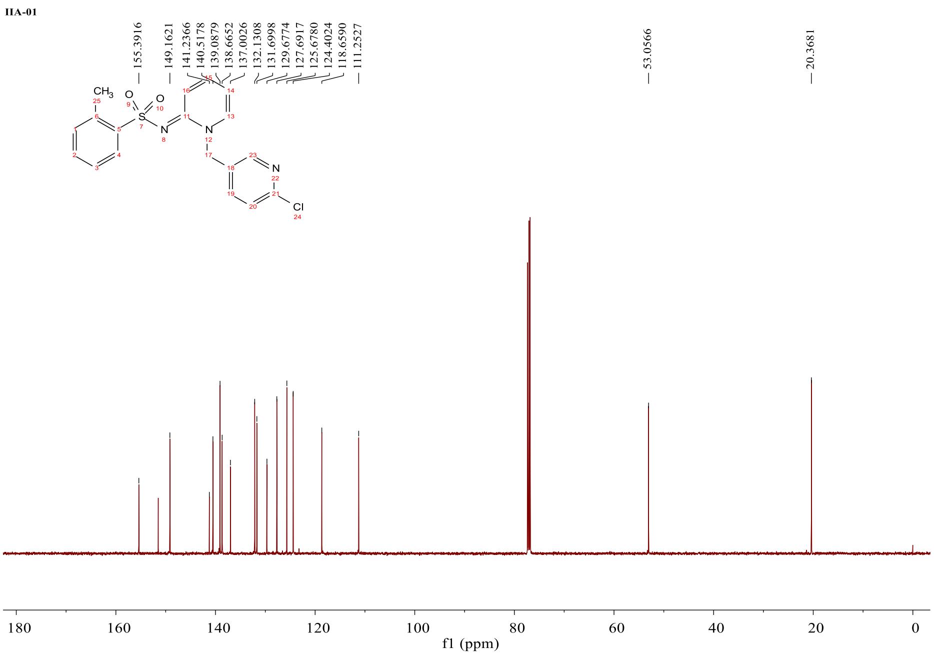

Figure S6. ^{13}C NMR of compound **2b.**

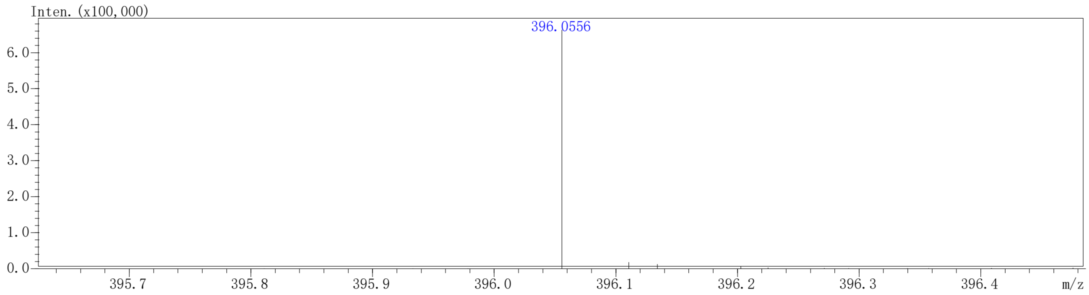

Figure S7. HRMS of compound **2b.**

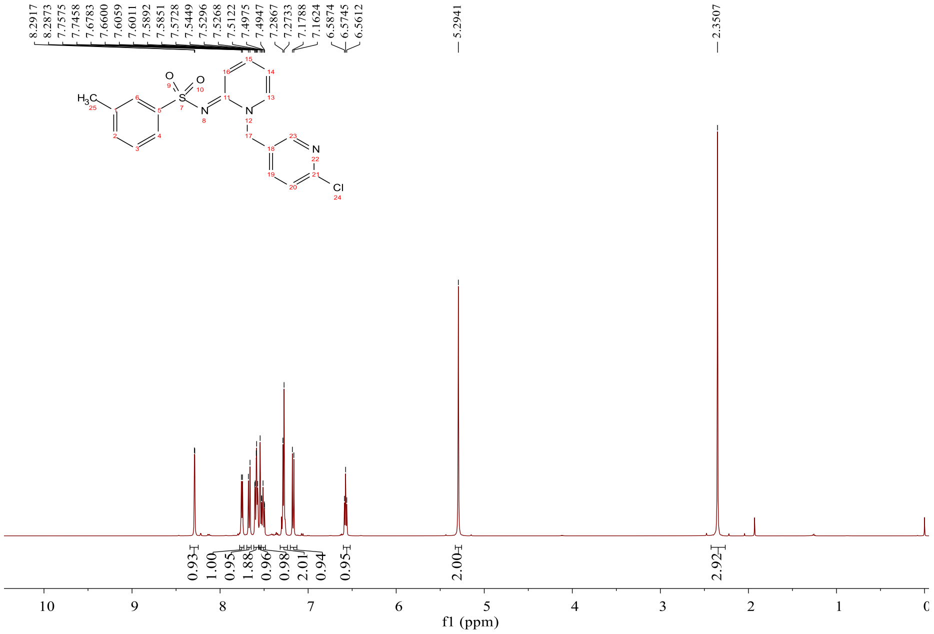

Figure S8. ^{1}H NMR of compound **2c.**

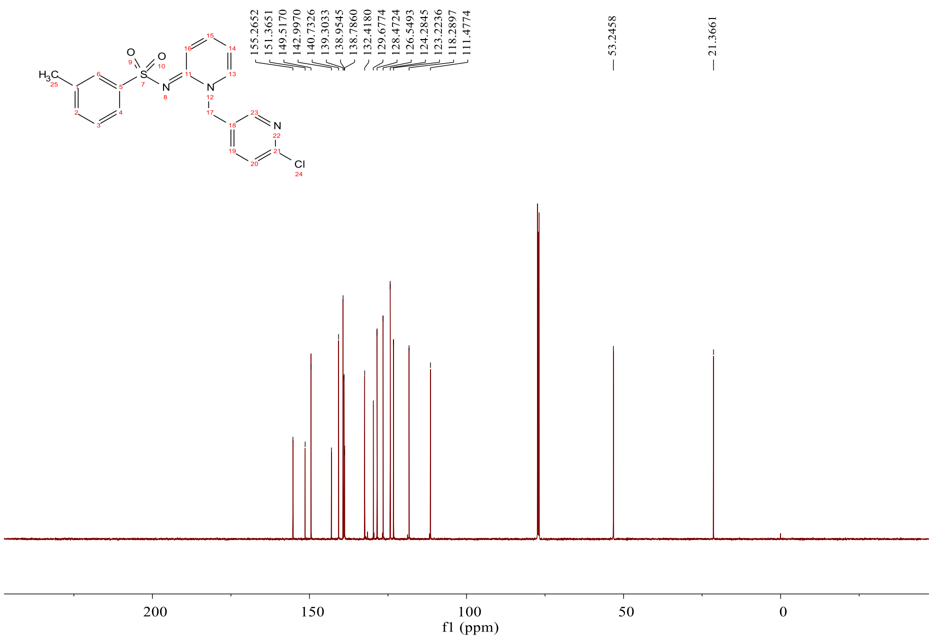

Figure S9. ^{13}C NMR of compound **2c.**

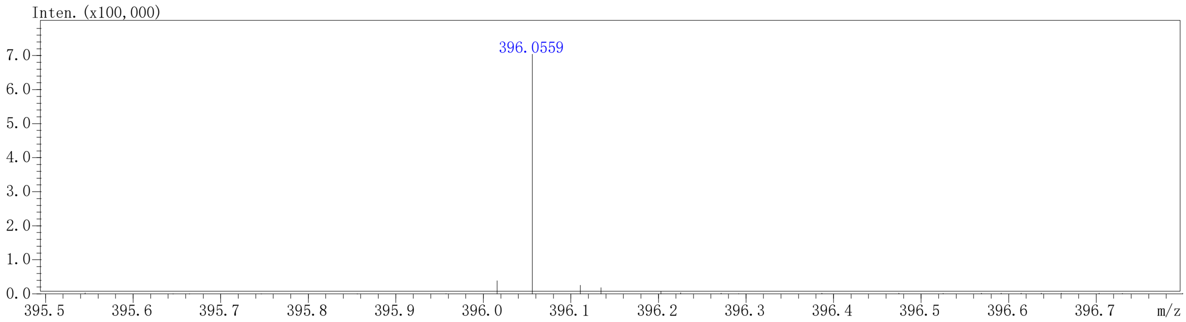

Figure S10. HRMS of compound **2c.**

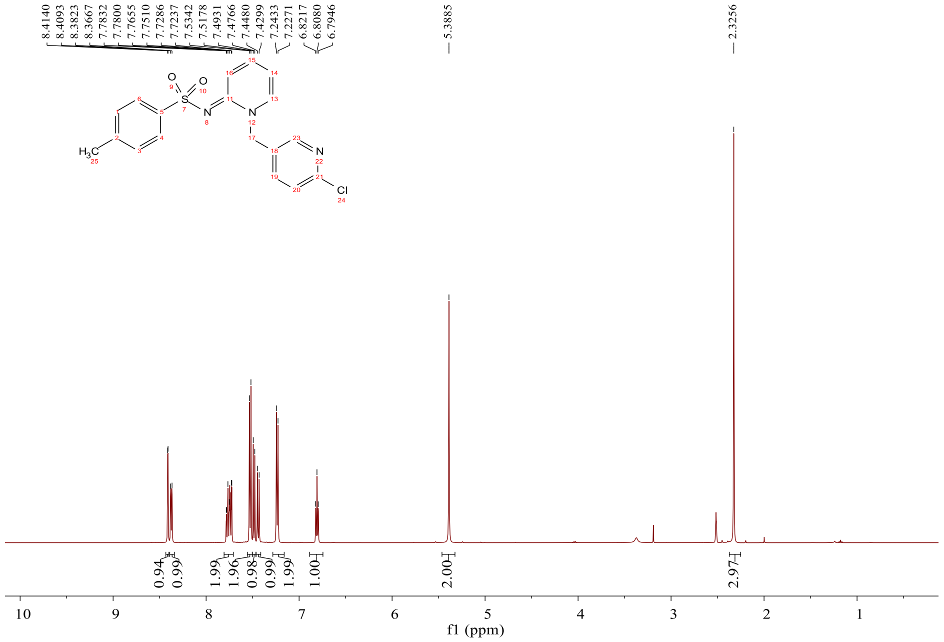

Figure S11. ^{1}H NMR of compound **2d.**

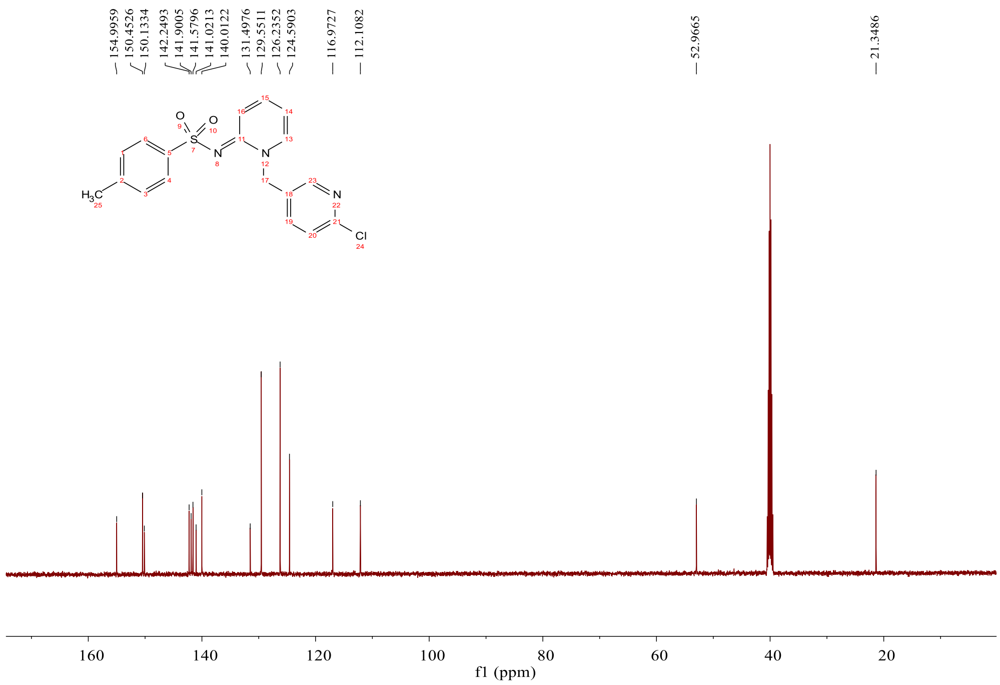

Figure S12. ^{13}C NMR of compound **2d.**

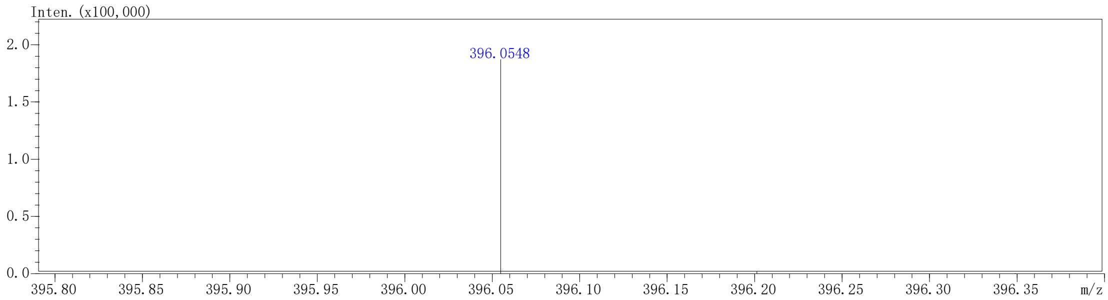

Figure S13. HRMS of compound **2d.**

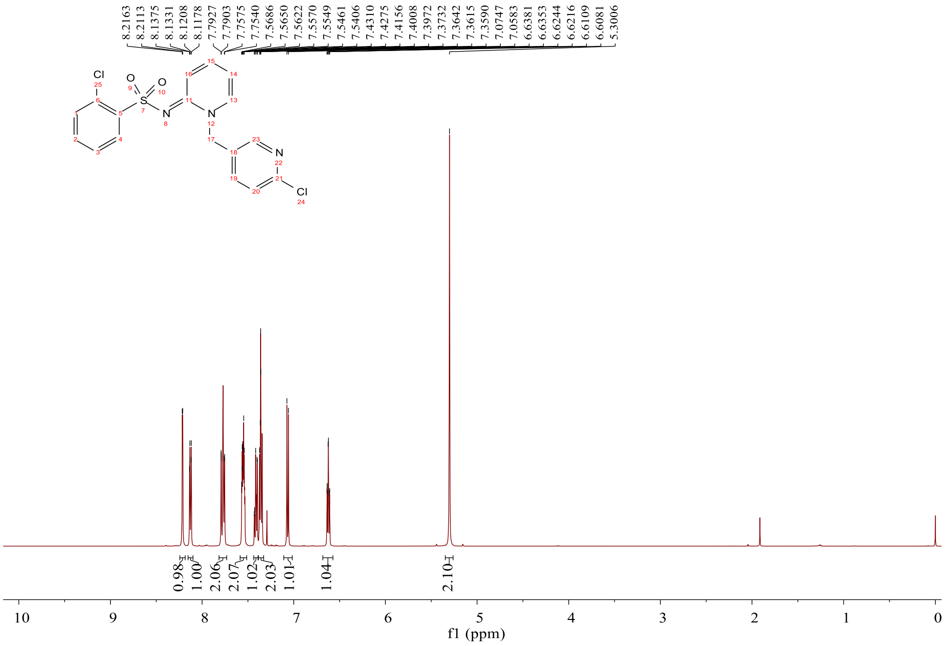

Figure S14. ^{1}H NMR of compound **2e.**

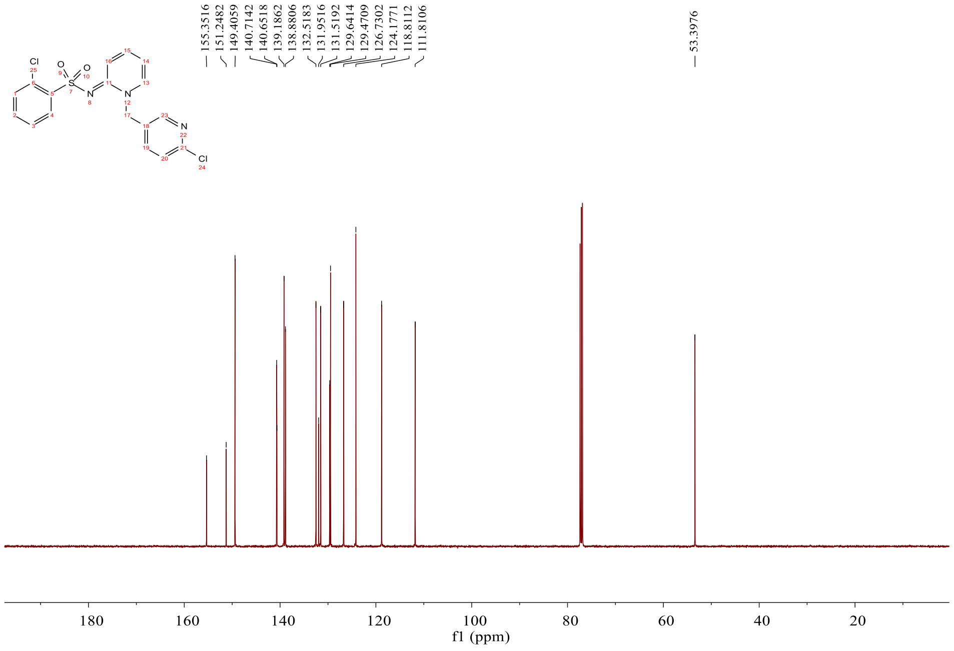

Figure S15. ^{13}C NMR of compound **2e.**

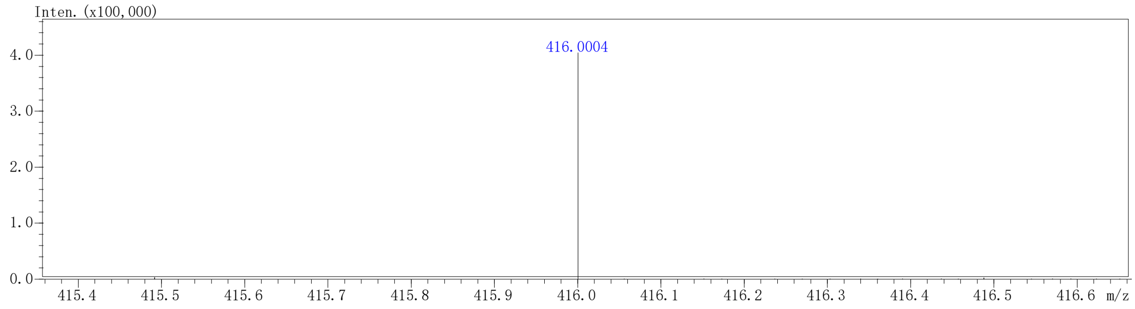

Figure S16. HRMS of compound **2e.**

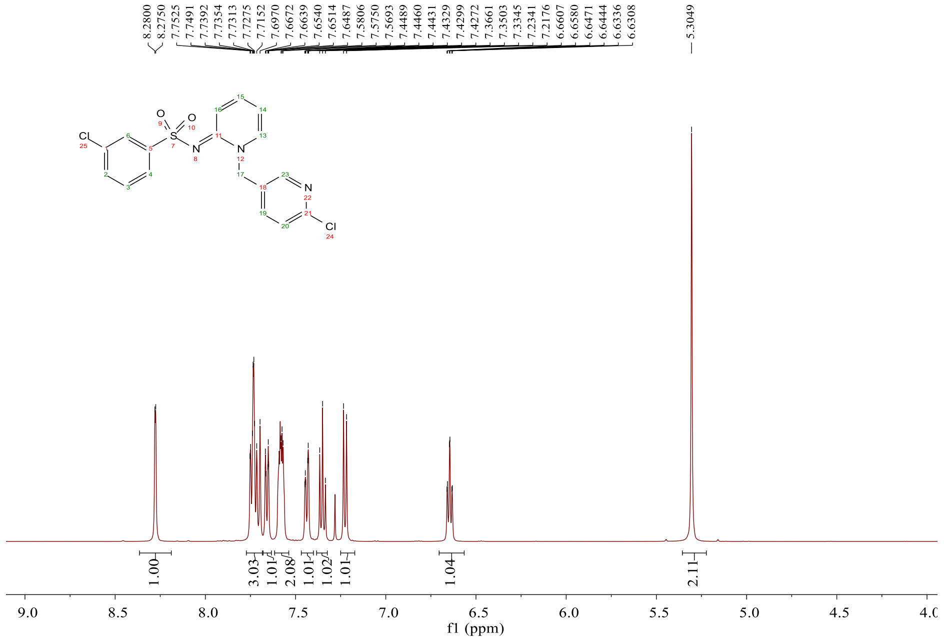

Figure S17. ^{1}H NMR of compound **2f.**

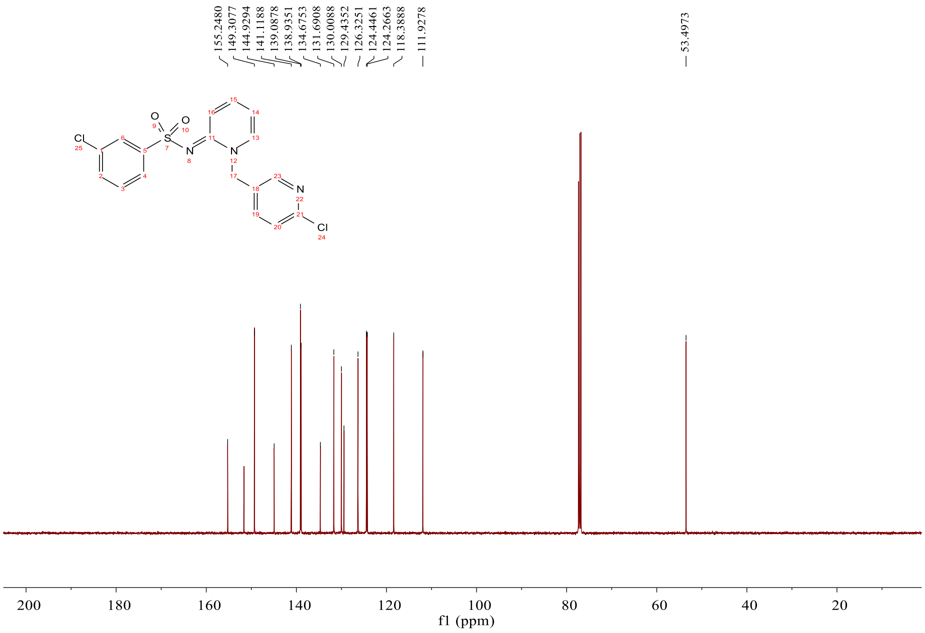

Figure S18. ^{13}C NMR of compound **2f.**

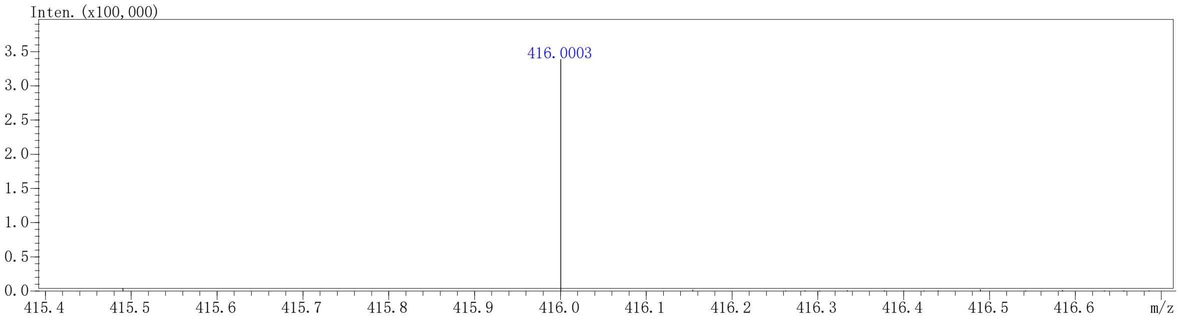

Figure S19. HRMS of compound **2f.**

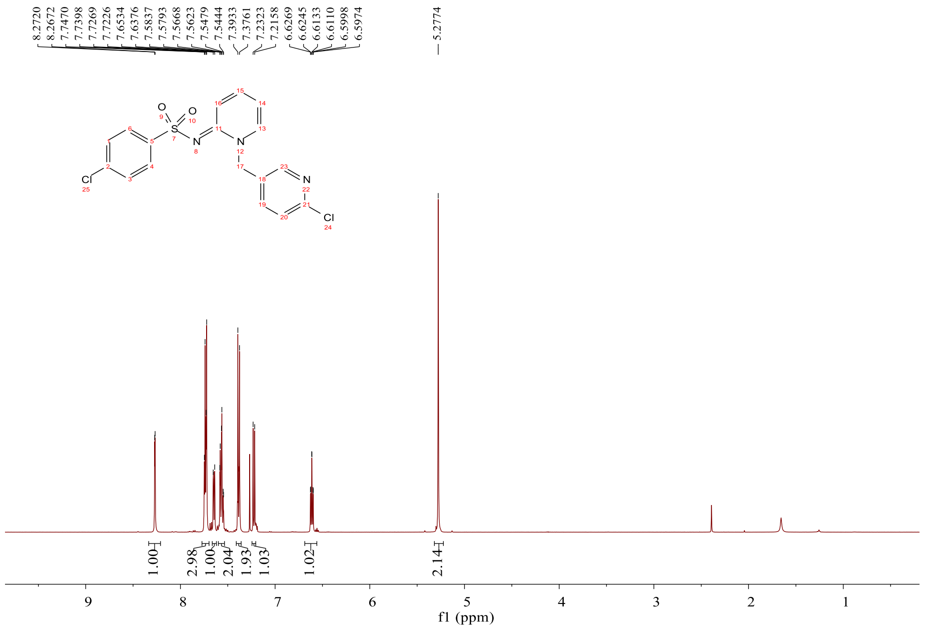

Figure S20. ^{1}H NMR of compound **2g.**

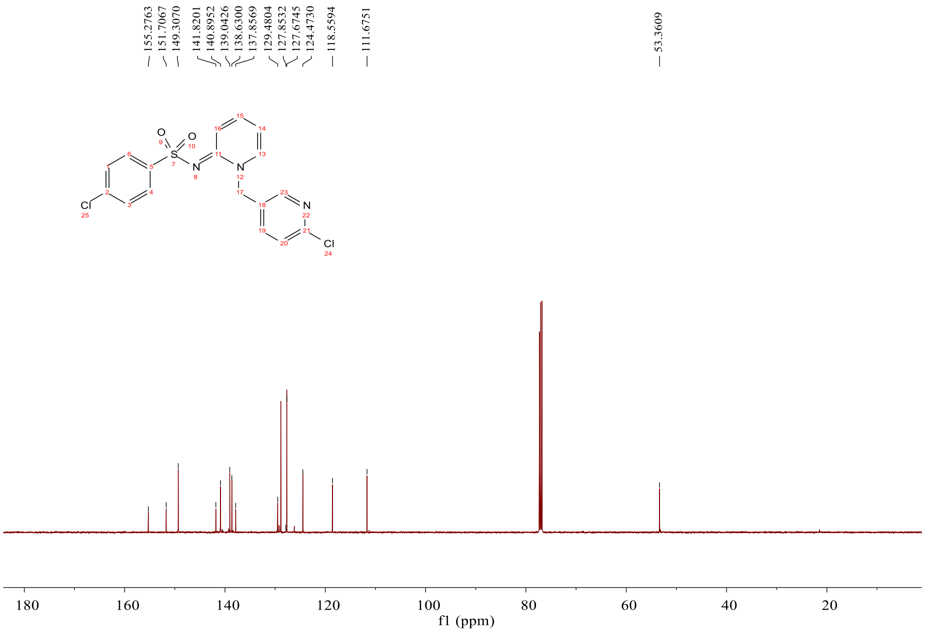

Figure S21. ^{13}C NMR of compound **2g.**

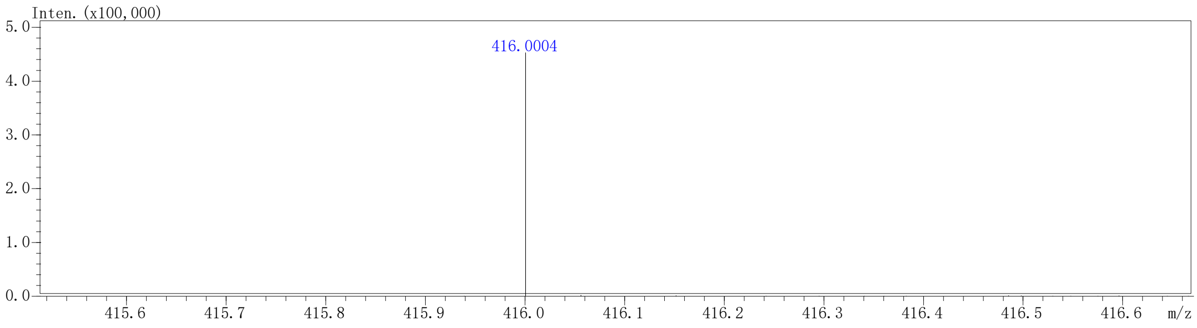

Figure S22. HRMS of compound **2g.**

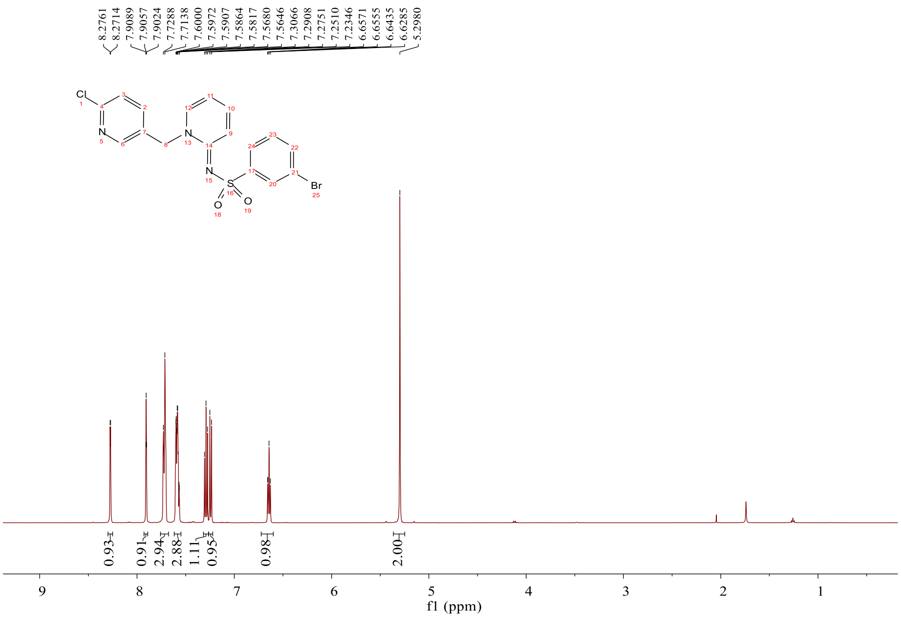

Figure S23. ^{1}H NMR of compound **2h.**

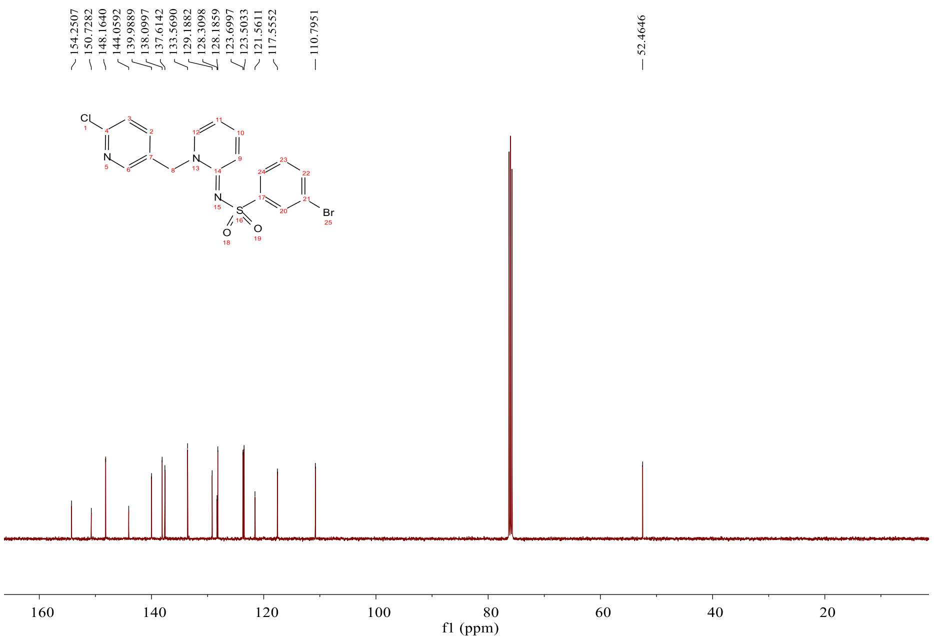

Figure S24. ^{13}C NMR of compound **2h.**

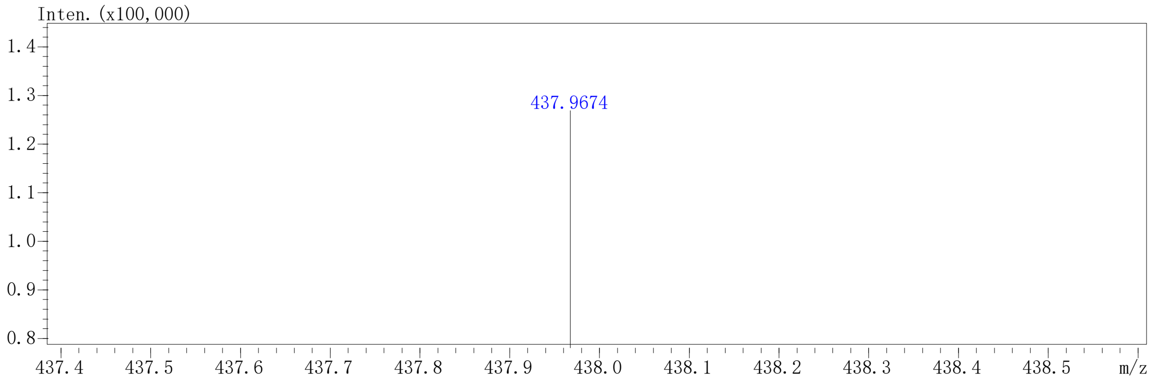

Figure S25. HRMS of compound **2h.**

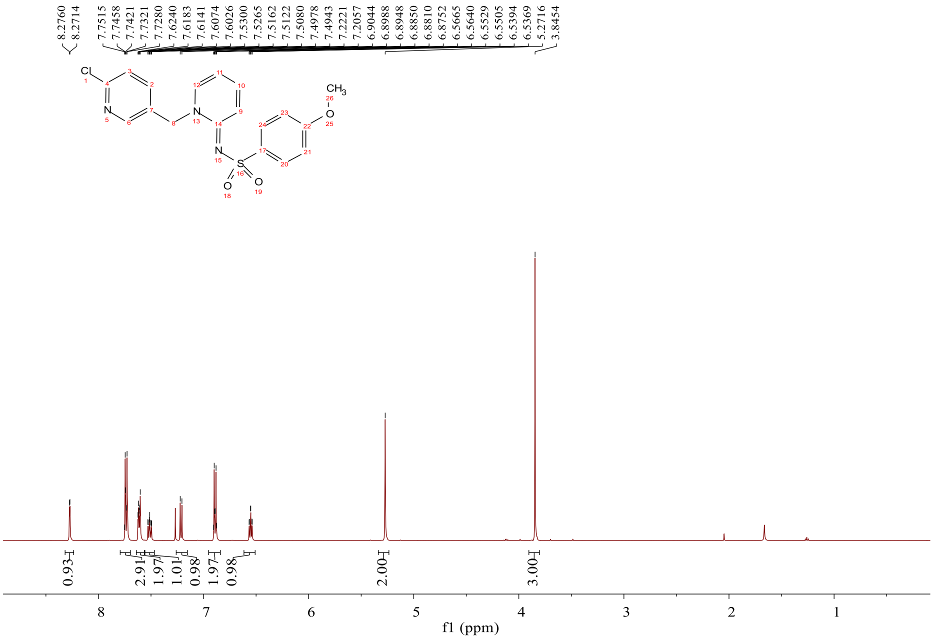

Figure S26. ^{1}H NMR of compound **2i.**

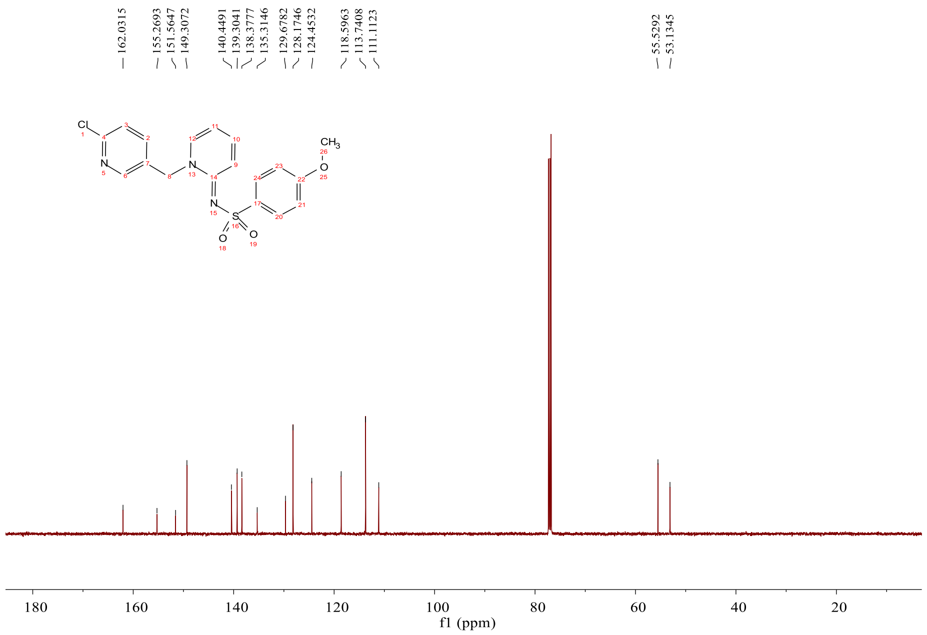

Figure S27. ^{13}C NMR of compound **2i.**

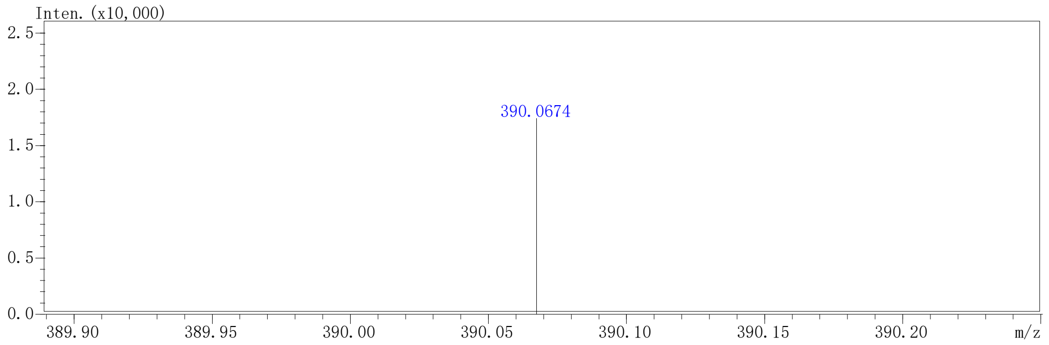

Figure S28. HRMS of compound **2i.**

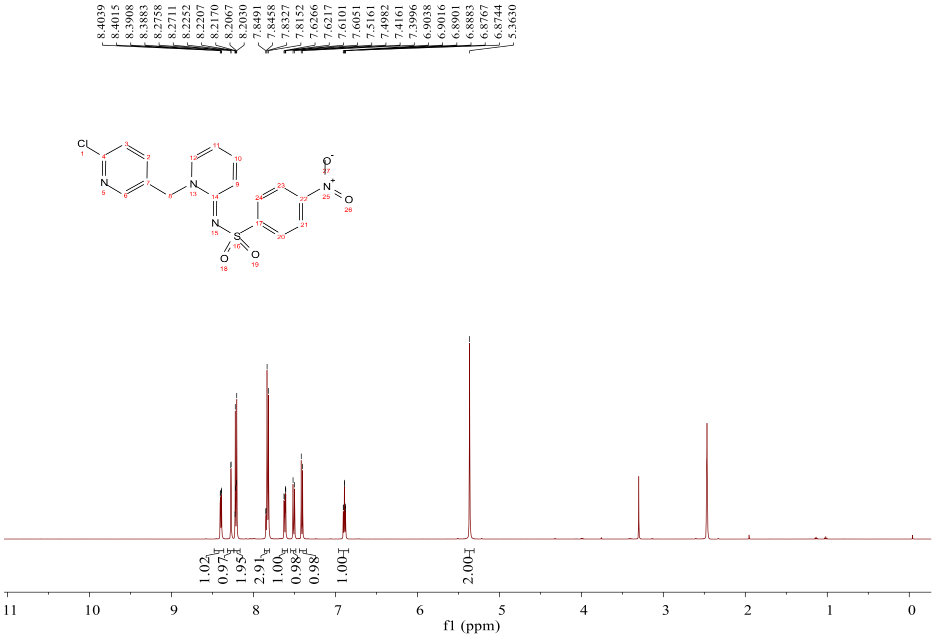

Figure S29. ^{1}H NMR of compound **2j.**

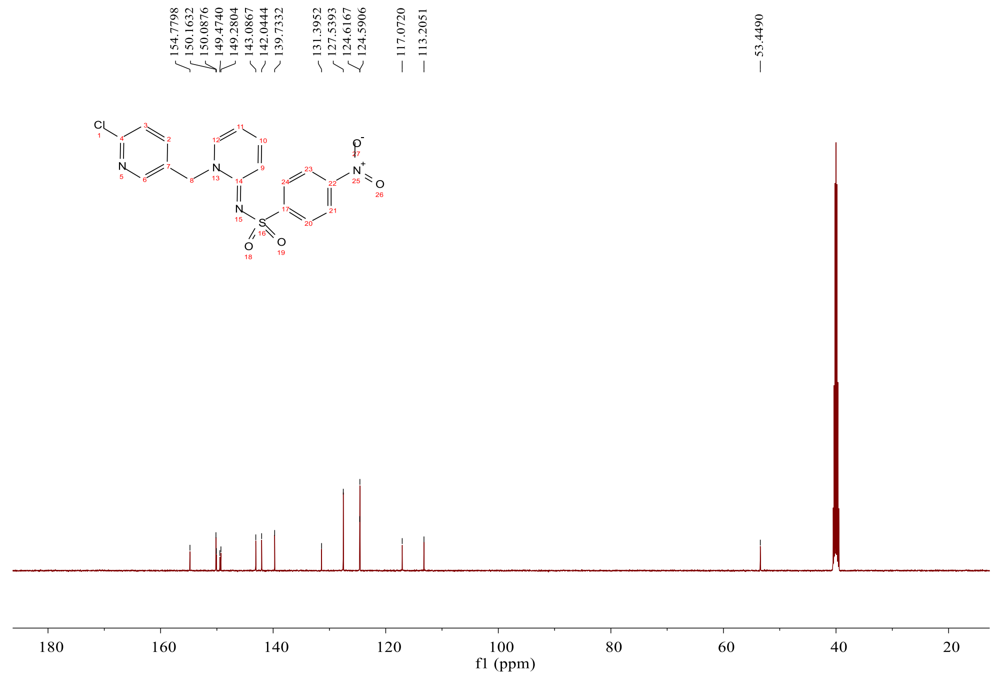

Figure S30. ^{13}C NMR of compound **2j.**

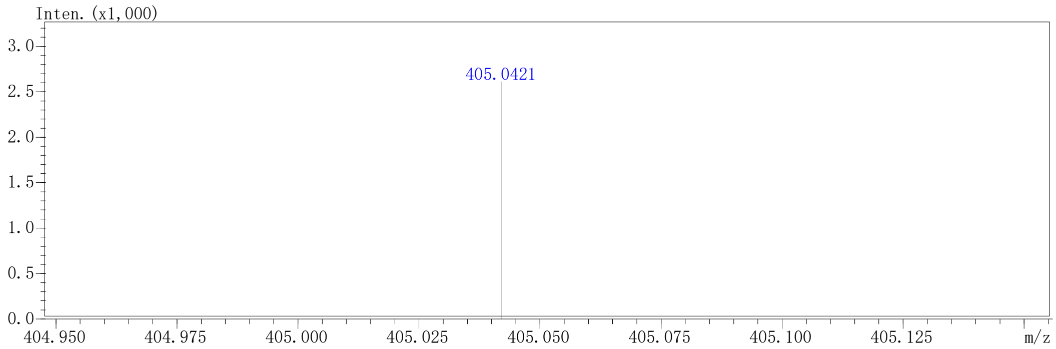

Figure S31. HRMS of compound **2j.**

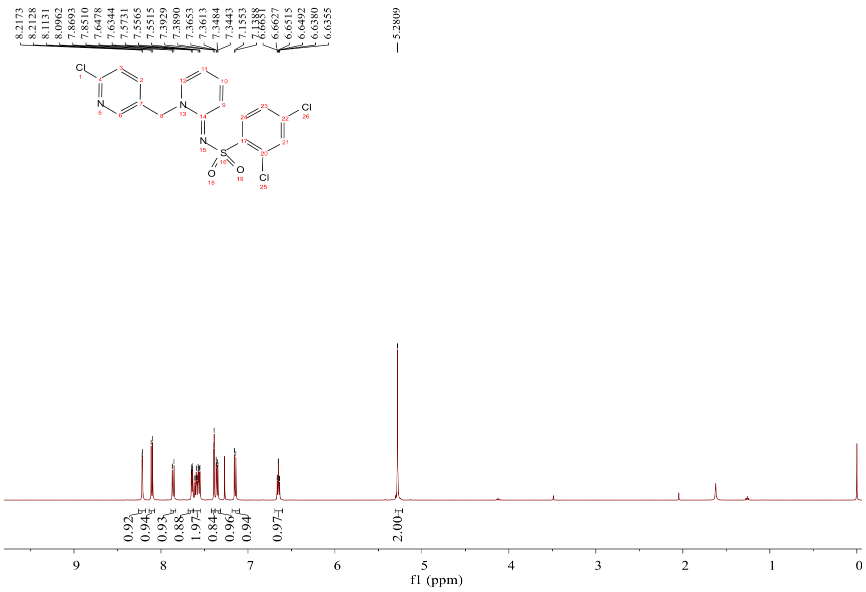

Figure S32. ^{1}H NMR of compound **2k.**

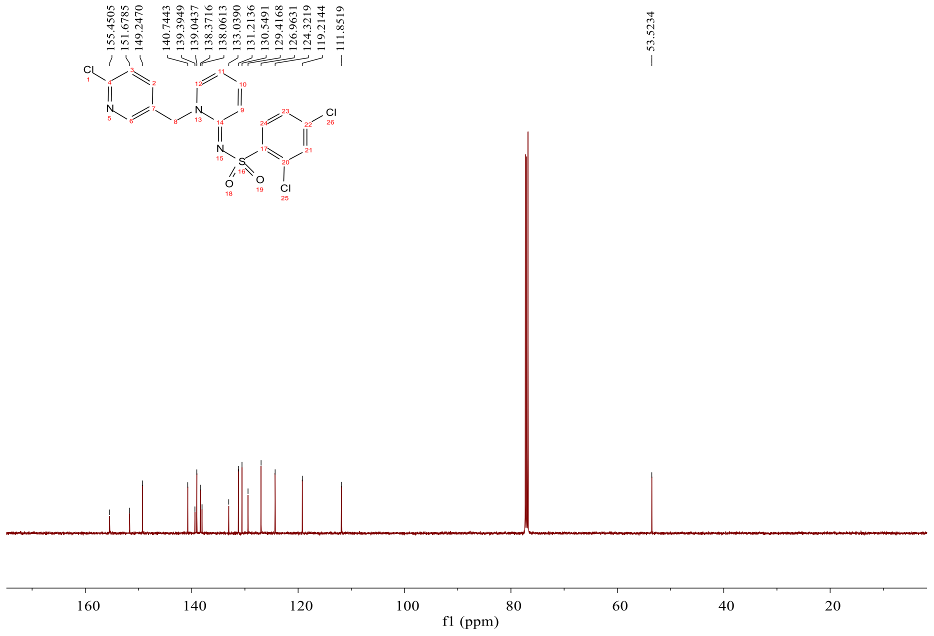

Figure S33. ^{13}C NMR of compound **2k.**

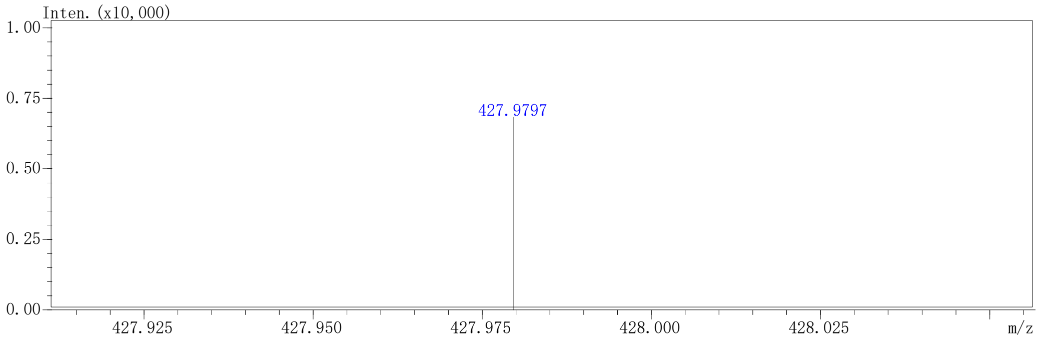

Figure S34. HRMS of compound **2k.**

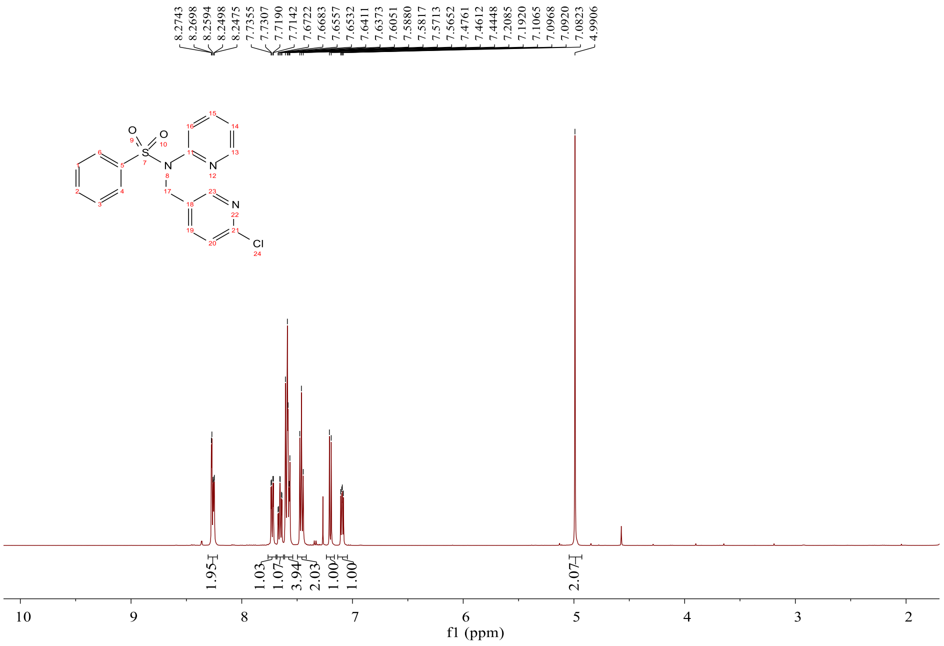

Figure S35. ^{1}H NMR of compound **3a.**

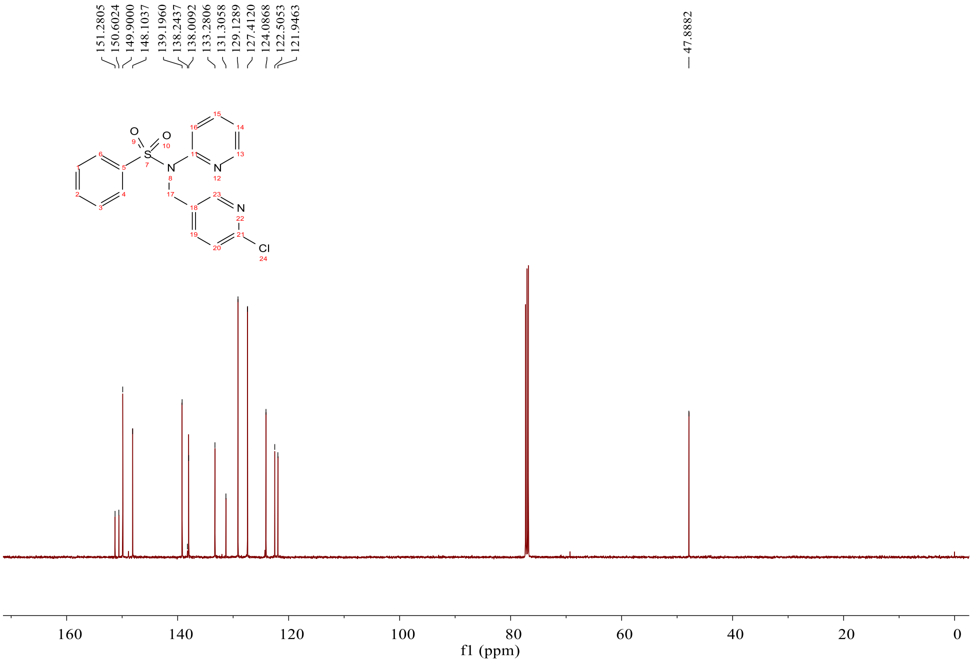

Figure S36. ^{13}C NMR of compound **3a.**

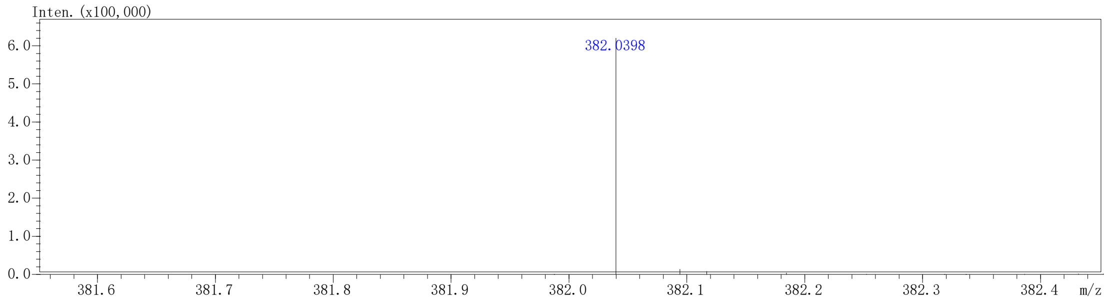

Figure S37. HRMS of compound **3a.**

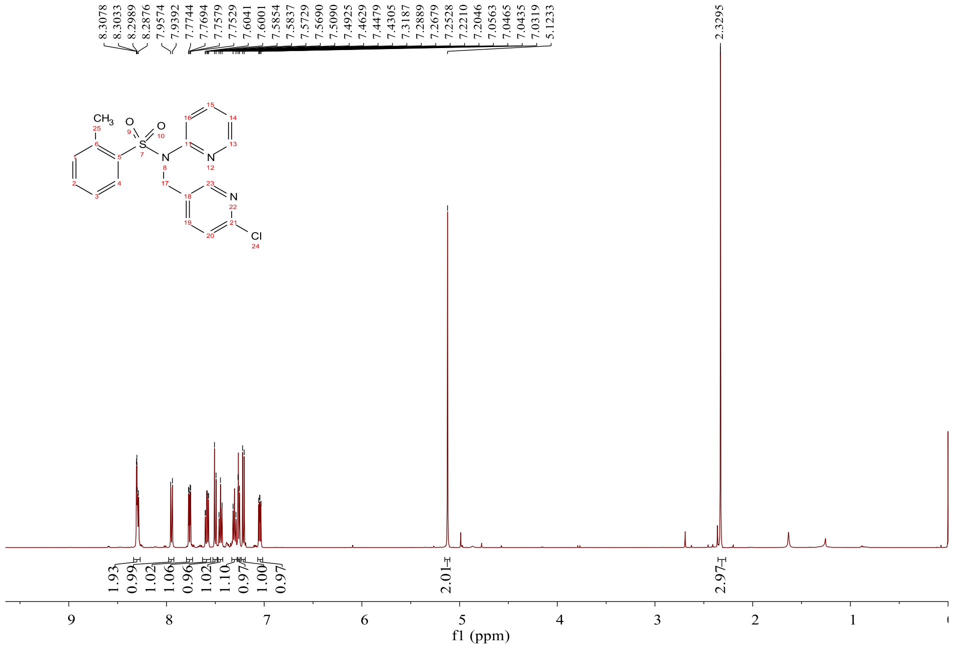

Figure S38. ^{1}H NMR of compound **3b.**

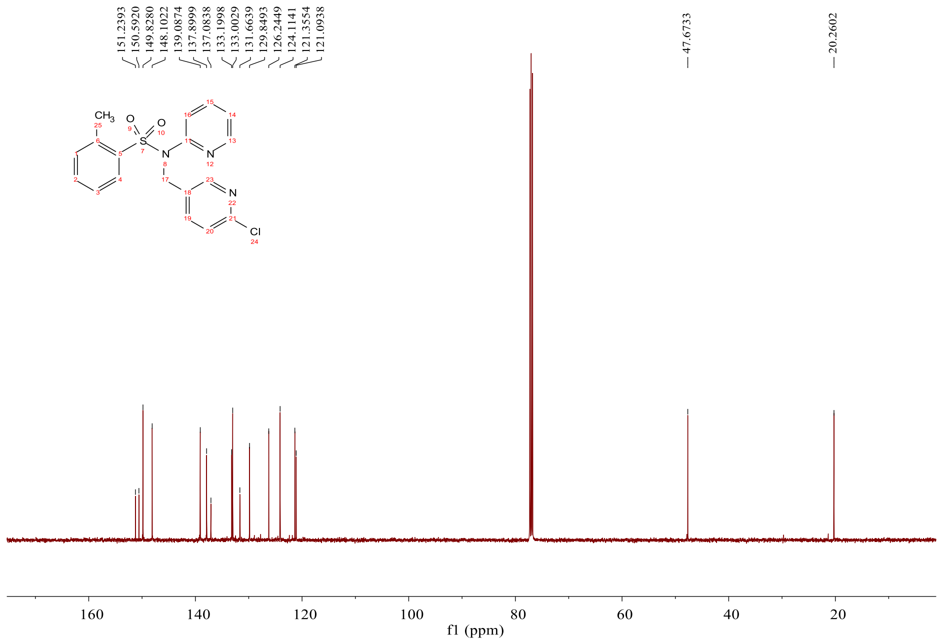

Figure S39. ^{13}C NMR of compound **3b.**

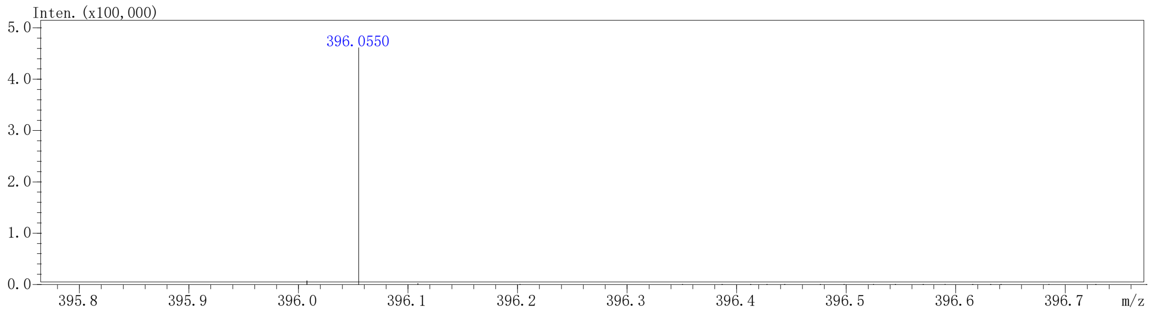

Figure S40. HRMS of compound **3b.**

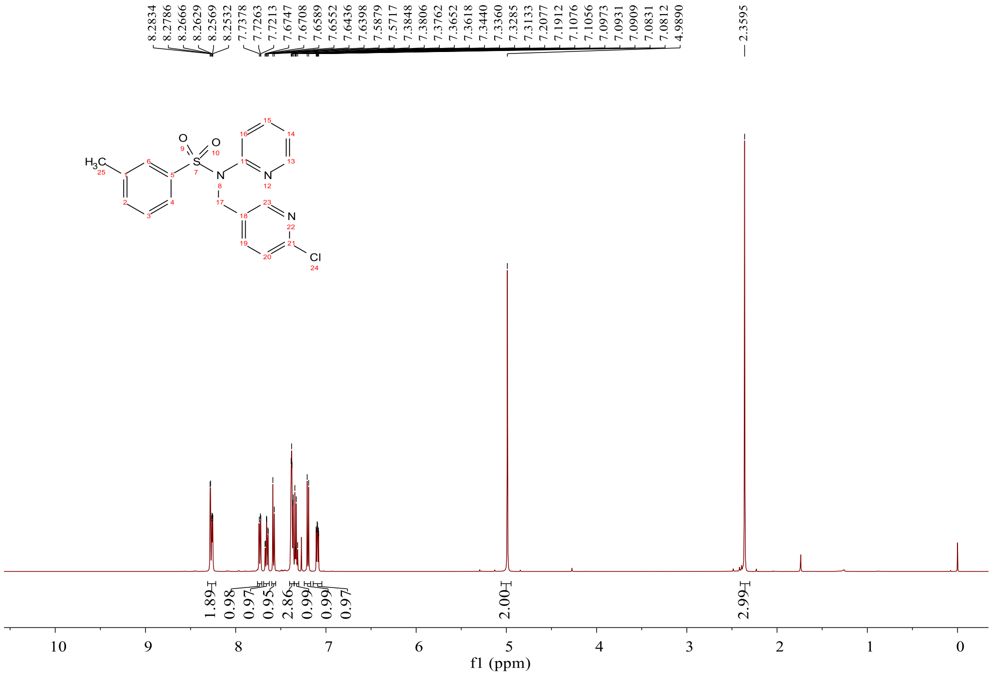

Figure S41. ^{1}H NMR of compound **3c.**

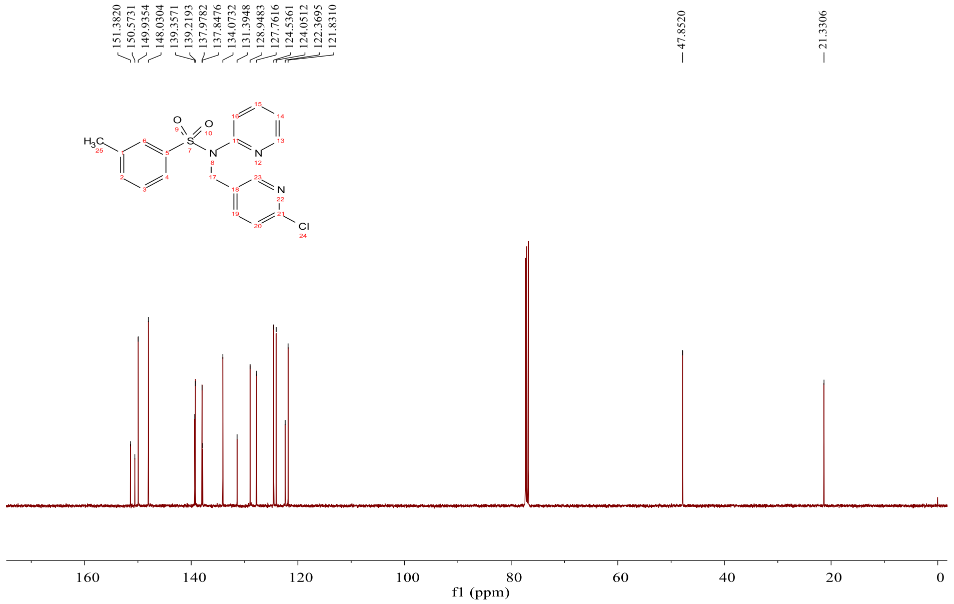

Figure S42. ^{13}C NMR of compound **3c.**

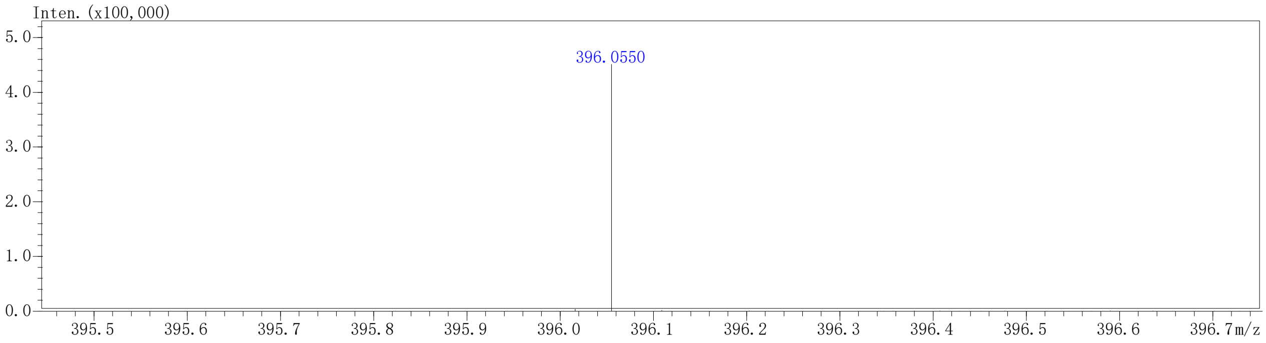

Figure S43. HRMS of compound **3c.**

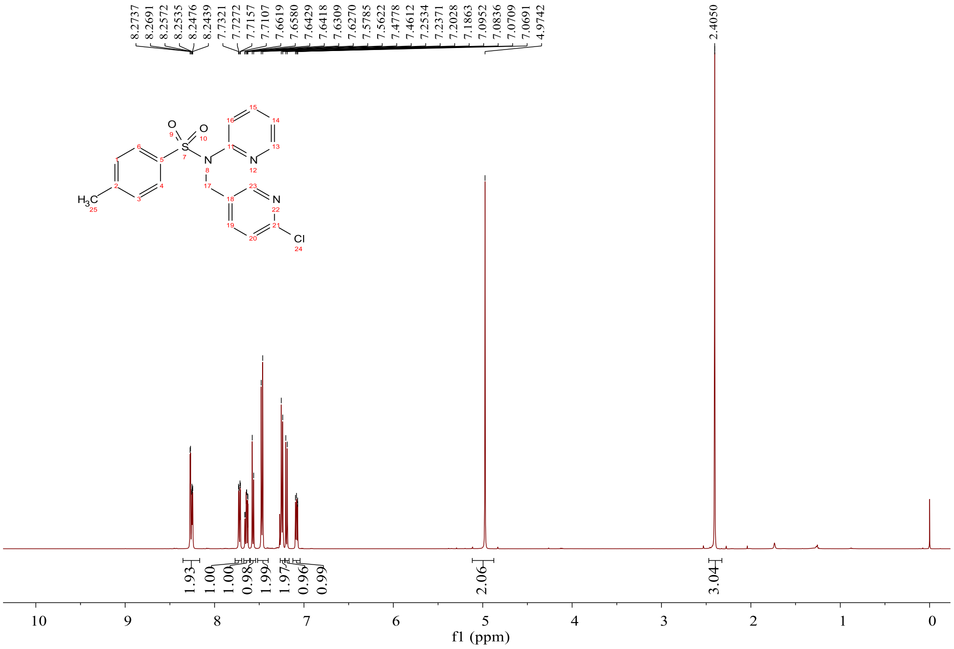

Figure S44. ^{1}H NMR of compound **3d.**

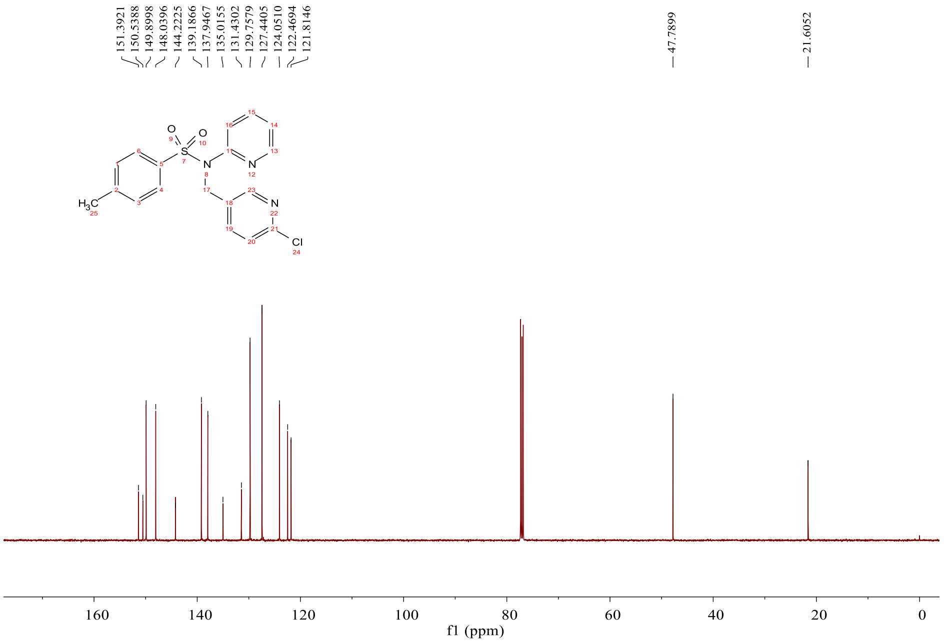

Figure S45. ^{13}C NMR of compound **3d.**

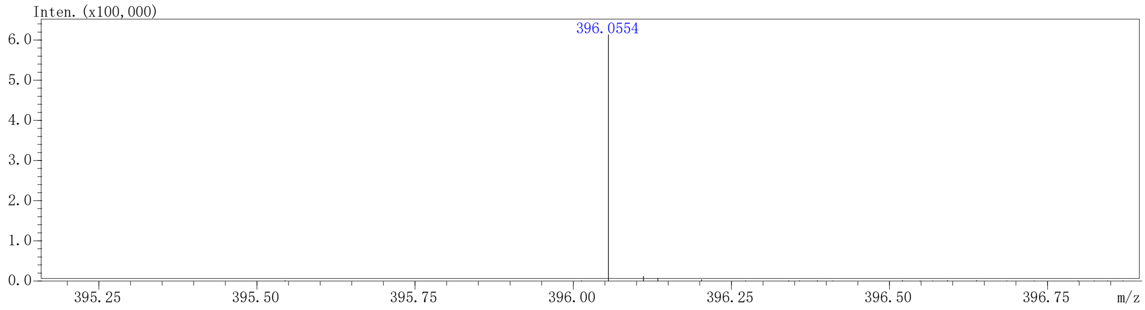

Figure S46. HRMS of compound **3d.**

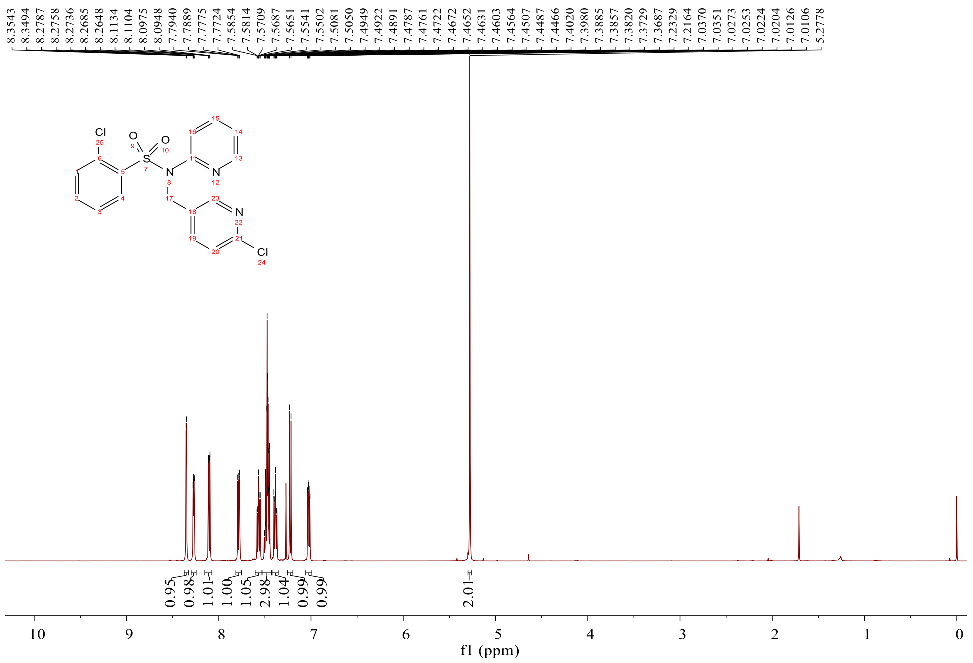

Figure S47. ^{1}H NMR of compound **3e.**

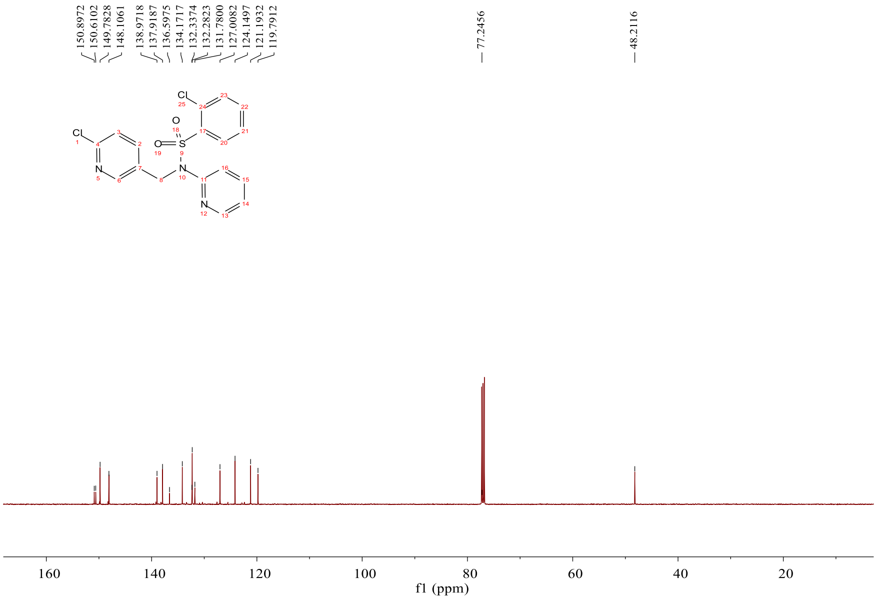

Figure S48. ^{13}C NMR of compound **3e.**

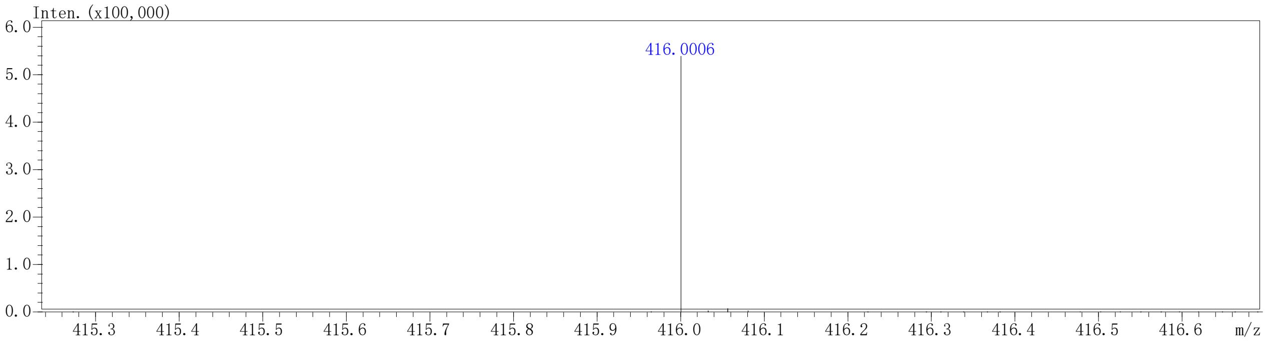

Figure S49. HRMS of compound **3e.**

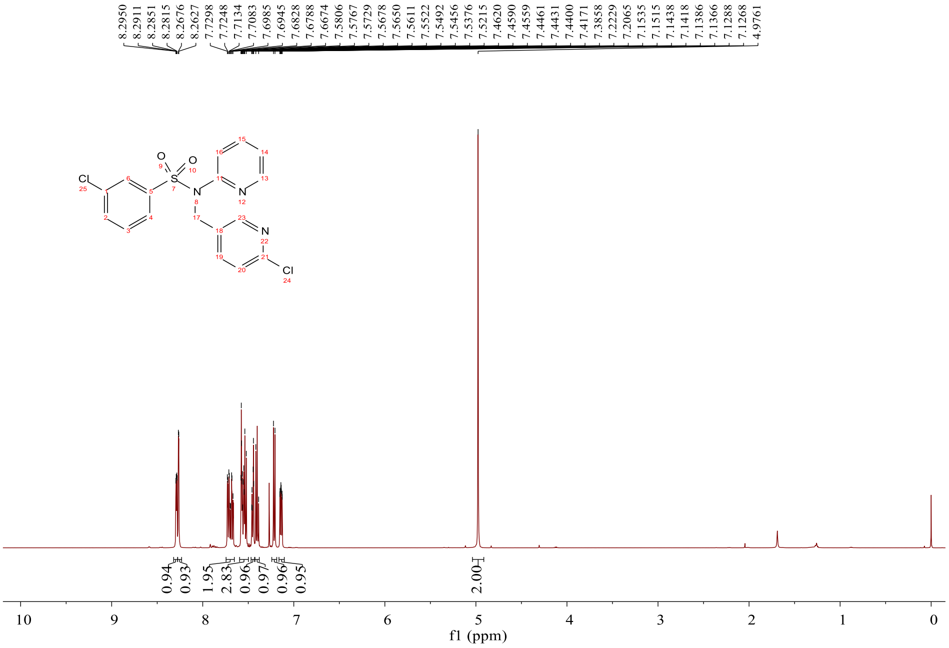

Figure S50. ^{1}H NMR of compound **3f.**

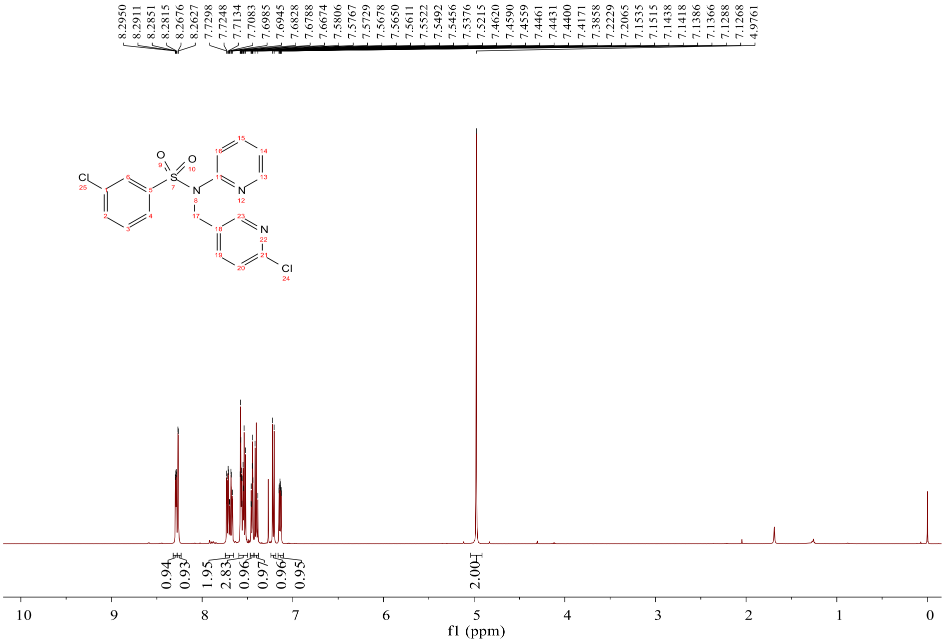

Figure S51. ^{13}C NMR of compound **3f.**

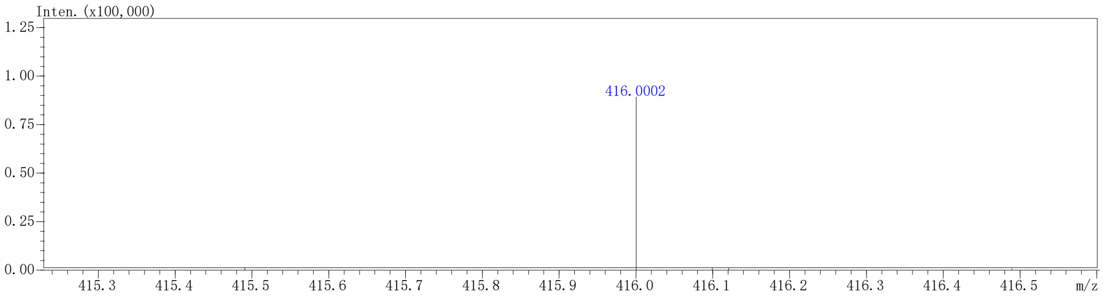

Figure S52. HRMS of compound **3f.**

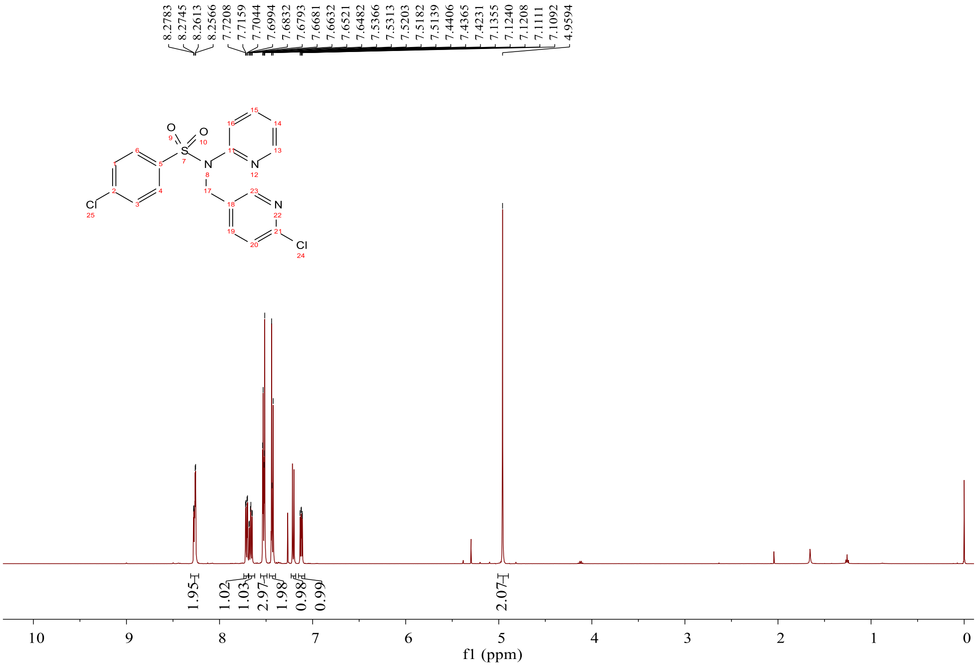

Figure S53. ^{1}H NMR of compound **3g.**

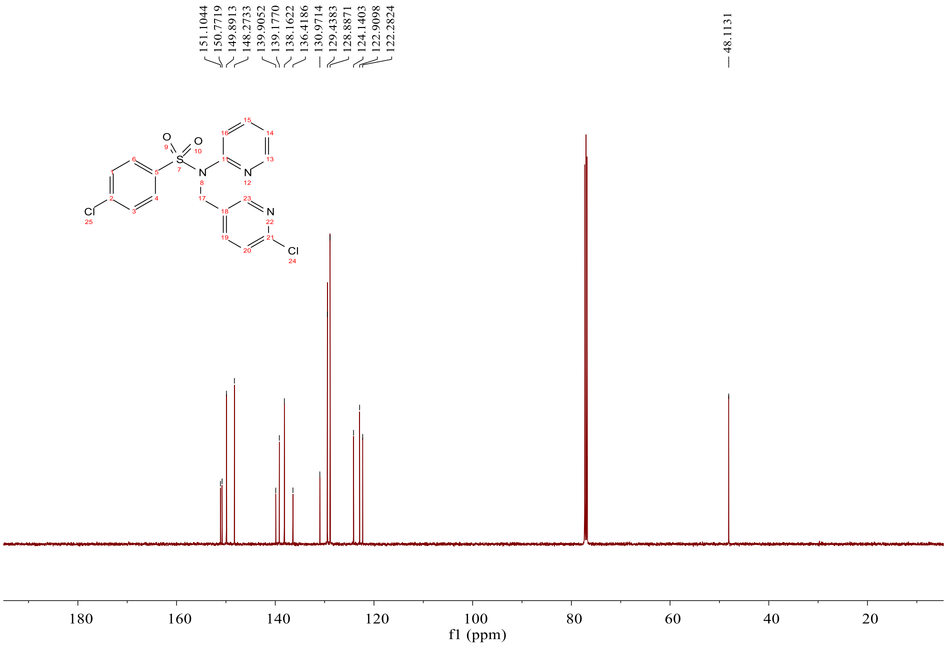

Figure S54. ^{13}C NMR of compound **3g.**

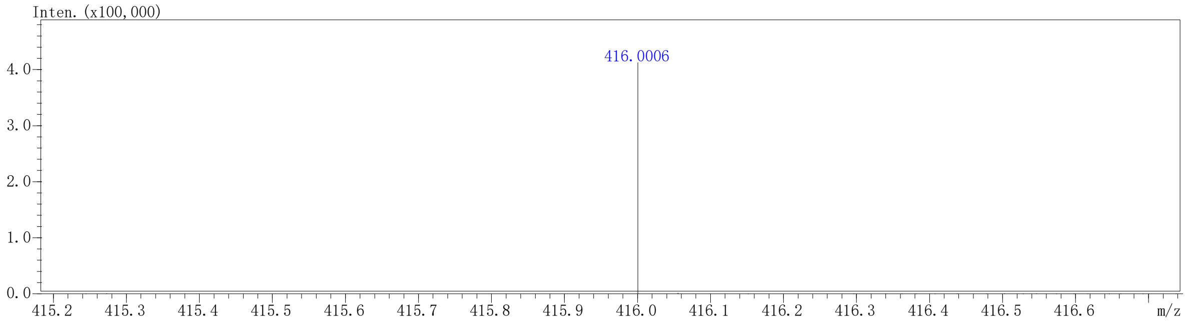

Figure S55. HRMS of compound **3g.**

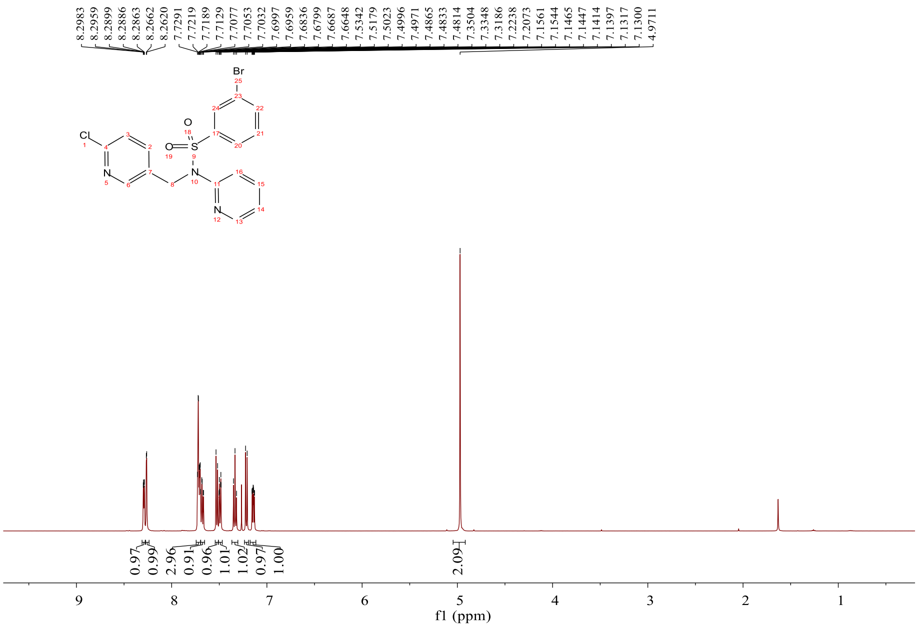

Figure S56. ^{1}H NMR of compound **3h.**

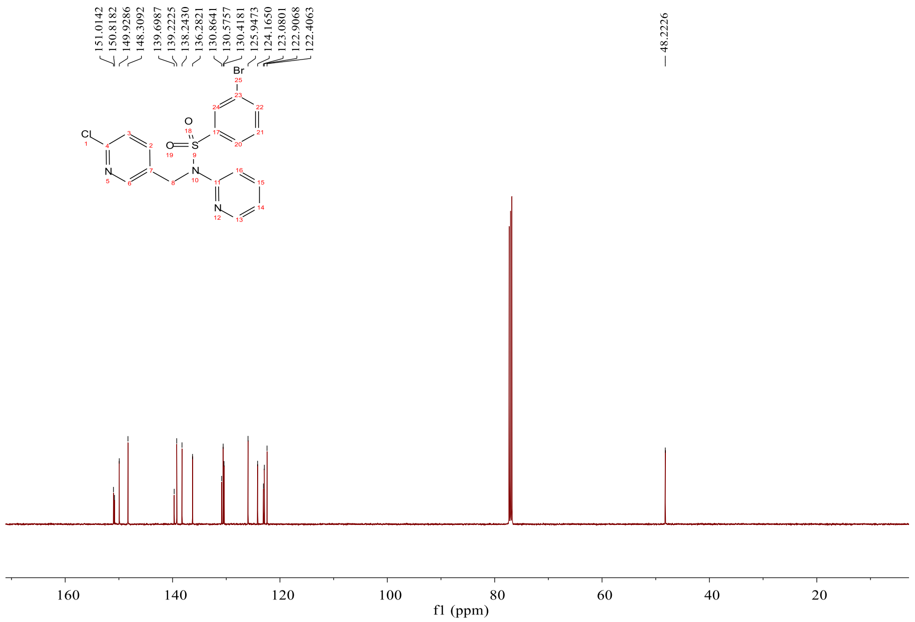

Figure S57. ^{13}C NMR of compound **3h.**

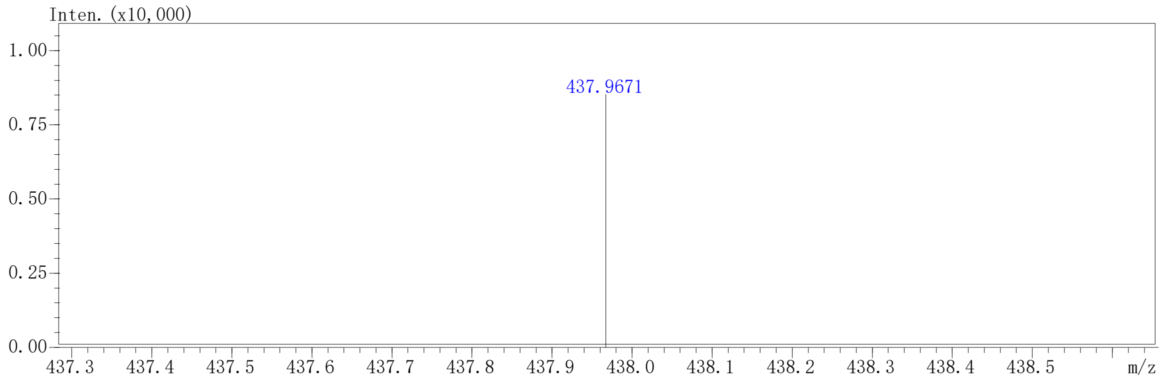

Figure S58. HRMS of compound **3h.**

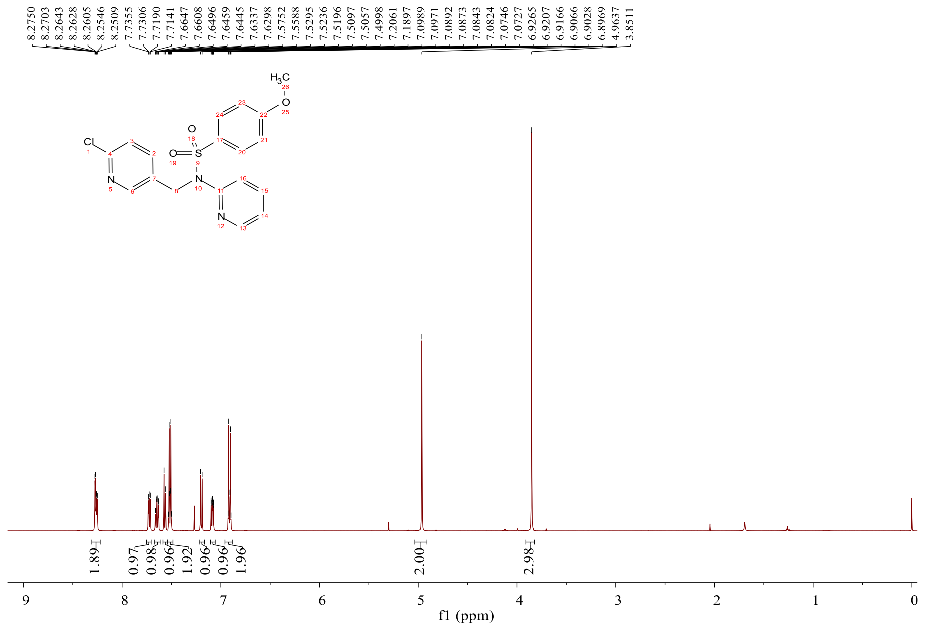

Figure S59. ^{1}H NMR of compound **3i.**

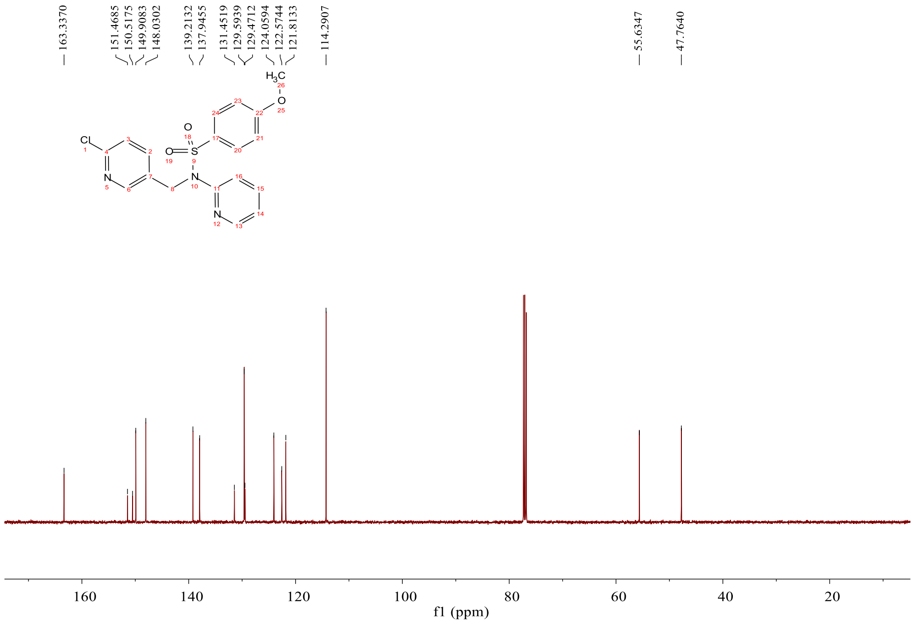

Figure S60. ^{13}C NMR of compound **3i.**

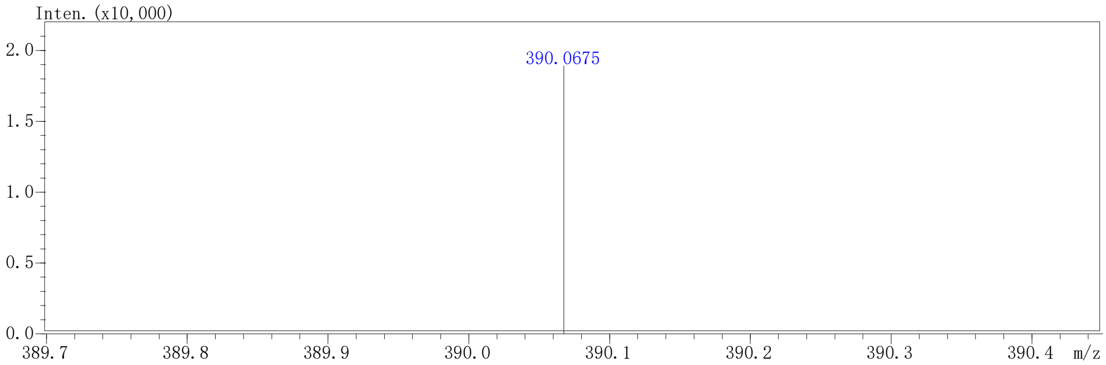

Figure S61. HRMS of compound **3i.**

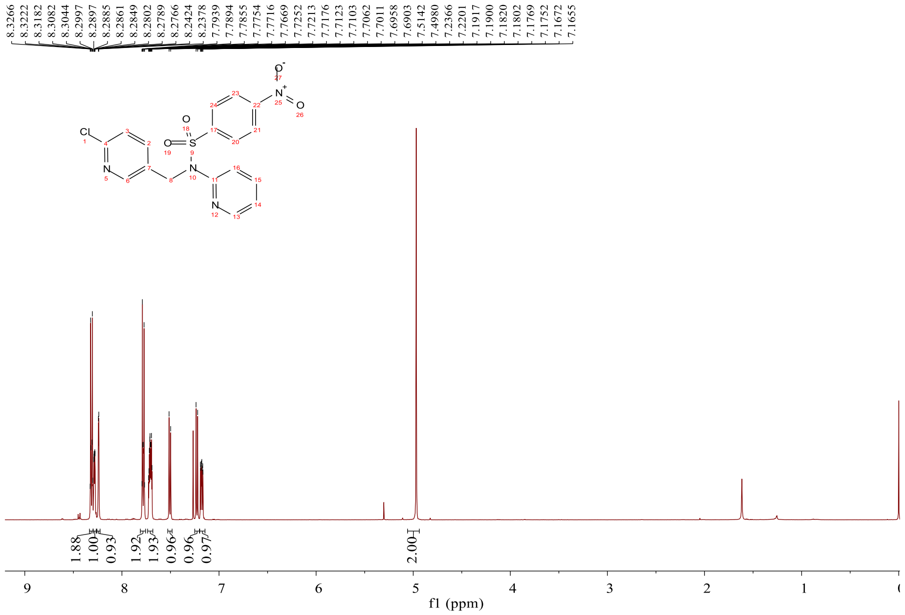

Figure S62. ^{1}H NMR of compound **3j.**

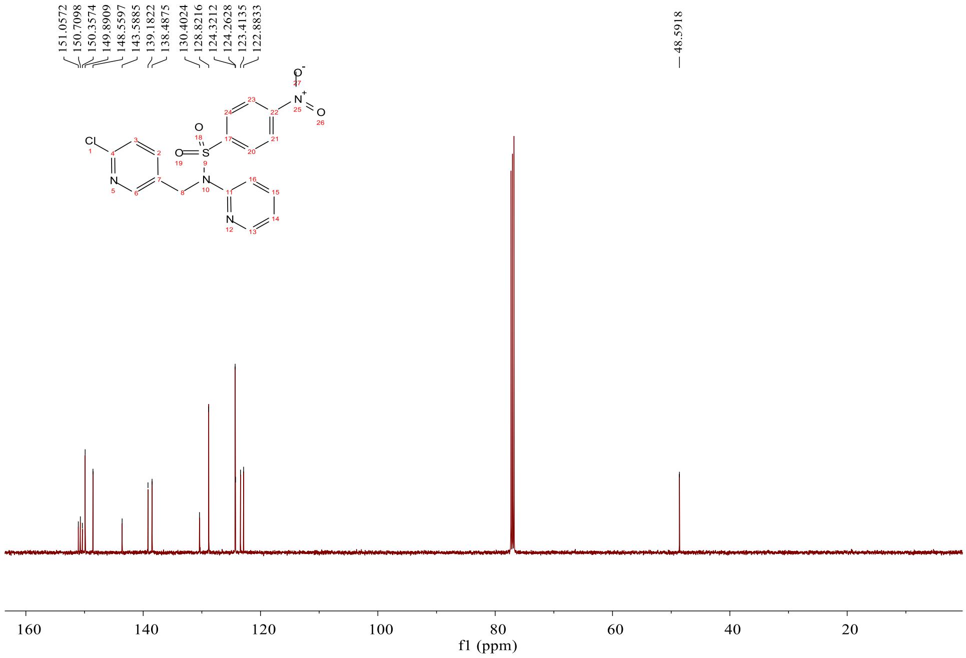

Figure S63. ^{13}C NMR of compound **3j.**

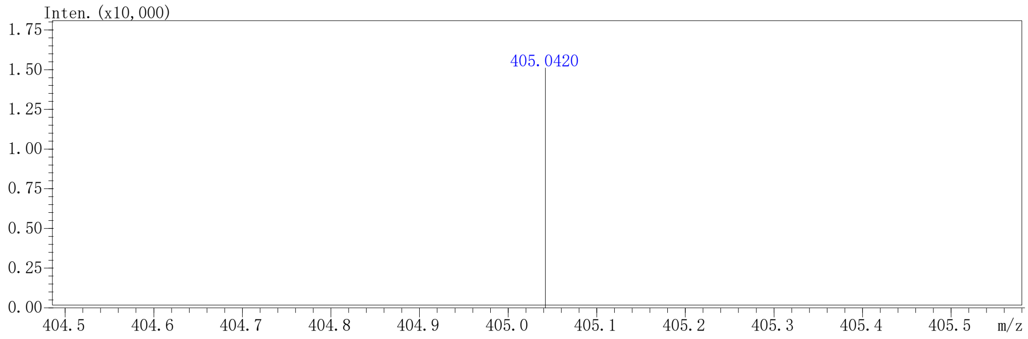

Figure S64. HRMS of compound **3j.**

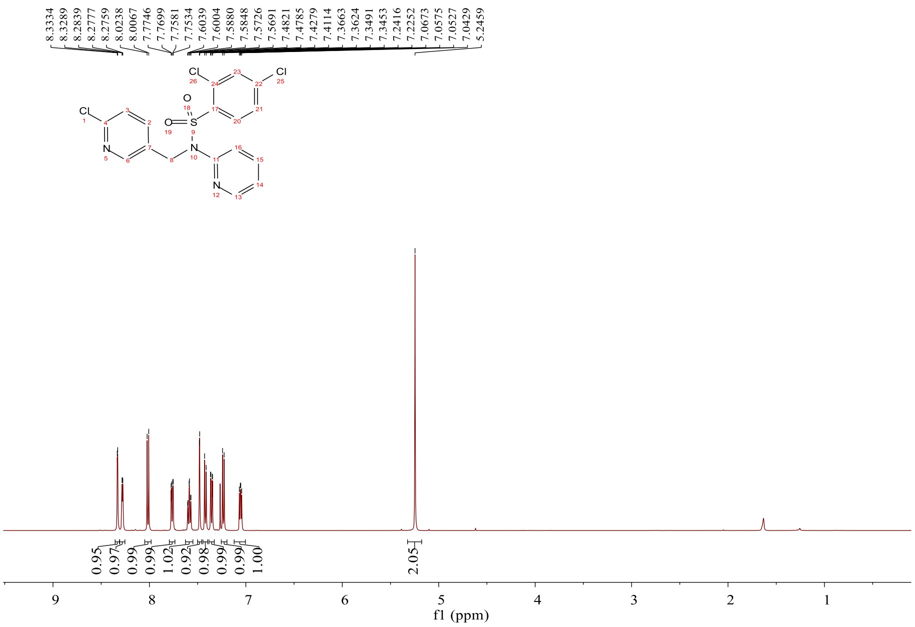

Figure S65. ^{1}H NMR of compound **3k.**

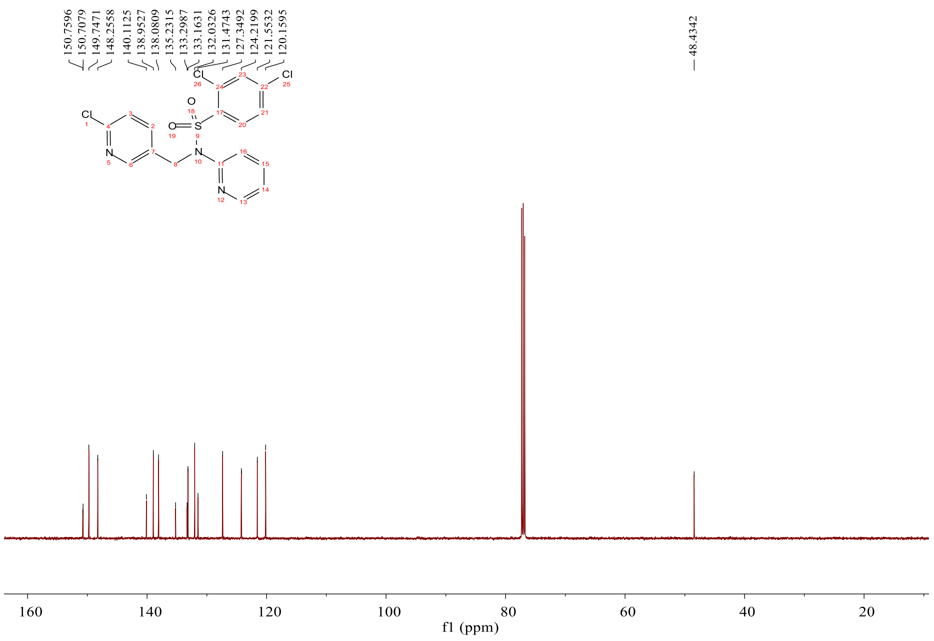

Figure S66. ^{13}C NMR of compound **3k.**

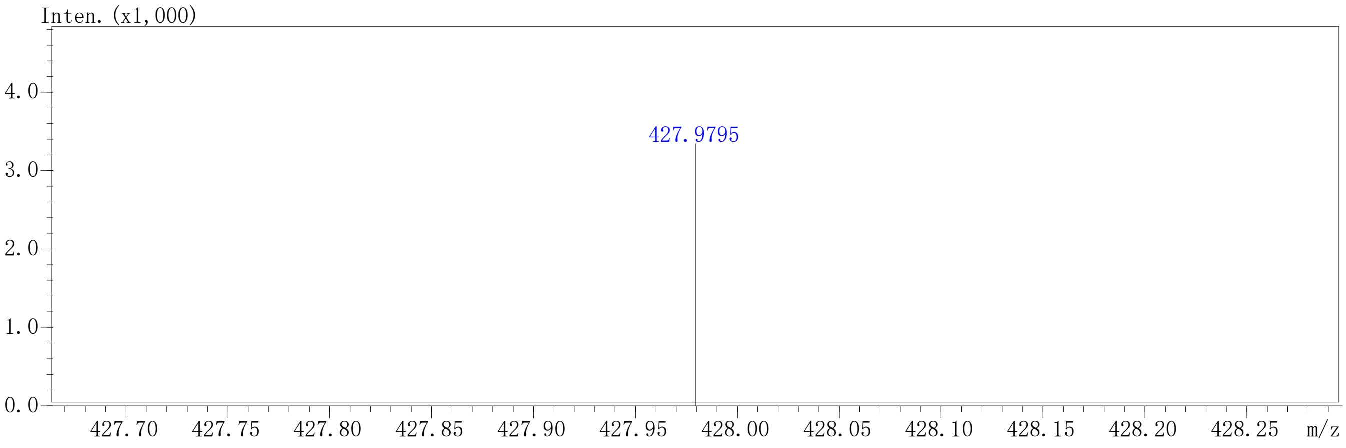

Figure S67. HRMS of compound **3k.**
